# Development of a Novel ^18^F-Labeled Radioligand for Imaging Phosphodiesterase 7 with Positron Emission Tomography

**DOI:** 10.1021/acs.molpharmaceut.4c01379

**Published:** 2025-02-19

**Authors:** Jian Rong, Chunyu Zhao, Ahmad F. Chaudhary, Evan Jones, Richard Van, Zhendong Song, Yinlong Li, Jiahui Chen, Xin Zhou, Jimmy S. Patel, Yabiao Gao, Zhenkun Sun, Siyan Feng, Zachary Zhang, Thomas L. Collier, Chongzhao Ran, Achi Haider, Yihan Shao, Hongjie Yuan, Steven H. Liang

**Affiliations:** † Department of Radiology and Imaging Sciences, Emory University, Atlanta, Georgia 30322, United States; ‡ Department of Chemistry and Biochemistry, University of Oklahoma, Norman, Oklahoma 73019, United States; § Department of Radiation Oncology, Winship Cancer Institute of Emory University, Atlanta, Georgia 30322, United States; ∥ Department of Pharmacology and Chemical Biology, Emory University School of Medicine, Atlanta, Georgia 30322, United States; ⊥ Athinoula A. Martinos Center for Biomedical Imaging, Department of Radiology, Massachusetts General Hospital and Harvard Medical School, Boston, Massachusetts 02114, United States

**Keywords:** phosphodiesterase 7, PDE7, [^18^F]P7−2302, PET, positron emission tomography

## Abstract

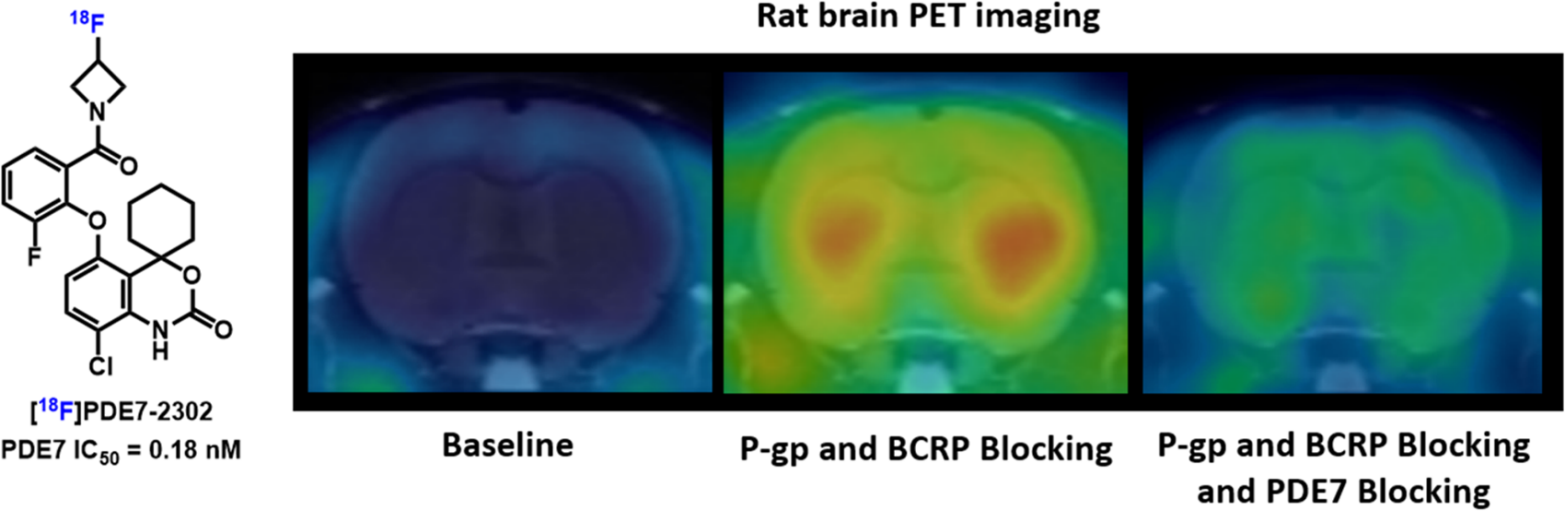

Phosphodiesterases (PDEs) are phosphohydrolytic enzymes responsible for degrading cyclic adenosine monophosphate (cAMP) and cyclic guanosine monophosphate (cGMP), two key second messengers involved in regulating cellular functions. The PDE superfamily can be subdivided into 11 families, with PDE7 playing a crucial role in the proinflammatory process, T-cell activation and proliferation. As such, PDE7 has emerged as a potential therapeutic target for treating inflammatory, immunological, and neurological disorders. To date, only a limited number of PDE7 PET ligands have been reported. These ligands often suffer from low *in vivo* stability or moderate binding affinity, underscoring the need for highly specific PET radioligands for imaging PDE7 *in vivo*. Here, we report the development of [^18^F]**7** ([^18^F]P7–2302**)–**a highly potent (IC_50_ = 0.18 nM) and selective (>400 folds over other PDEs) PDE7 PET ligand. *In vitro* autoradiography studies using rat brain sections revealed high PDE7-specific binding for [^18^F]**7**. Notwithstanding these encouraging findings, PET imaging experiments in rats demonstrated low brain uptake of [^18^F]**7**, potentially owing to brain efflux mechanism. Indeed, *in vivo* studies with combined P-gp and BCRP inhibition substantially improved brain uptake and enabled us to demonstrate *in vivo* binding specificity of [^18^F]**7** with PDE**7-**targeted blockade. Overall, [^18^F]**7** ([^18^F]P7–2302**)** exhibits promising pharmacological properties and chemical scaffold which holds potential as a PDE7-specific PET radioligand, though further work is required to enhance blood-brain barrier permeability.

## Introduction

Phosphodiesterases (PDEs) are phosphohydrolytic enzymes that degrade cyclic adenosine monophosphate (cAMP) and cyclic guanosine monophosphate (cGMP)—two pivotal second messenger molecules that orchestrate essential cellular functions. PDEs are key regulators of signal transduction in immune cells, neurons, and cardiovascular systems.^1,2^ The PDE superfamily is categorized into 11 subfamilies (PDE1–11) based on the sequence homology of their C-terminal catalytic domains, and these are further classified by substrate specificity into cAMP-specific (PDE4, PDE7, and PDE8), cGMP-specific (PDE5, PDE6, and PDE9), and dual-specific PDEs (PDE1, PDE2, PDE3, PDE10, and PDE11).

The PDE7 subfamily consists of two isoforms, PDE7A and PDE7B. While PDE7A is widely expressed in the central nervous system (CNS) and peripheral organs,^3,4^ PDE7B is mainly expressed in the CNS, particularly in regions such as the striatum and thalamus.^4,5^ PDE7 plays a significant role in driving proinflammatory processes, as showcased by its role in T-cell activation and proliferation.^6^ PDE7 is the potential therapeutic target for treating inflammatory, immunological disorders, and CNS diseases.^7^ Previous research revealed that inhibition of PDE7 offers therapeutic benefits for neurodegenerative diseases,^8,9^ including Alzheimer’s disease (AD),^10,11^ Parkinson’s disease (PD),^12−14^ and multiple sclerosis (MS).^15,16^

**Figure 1:**
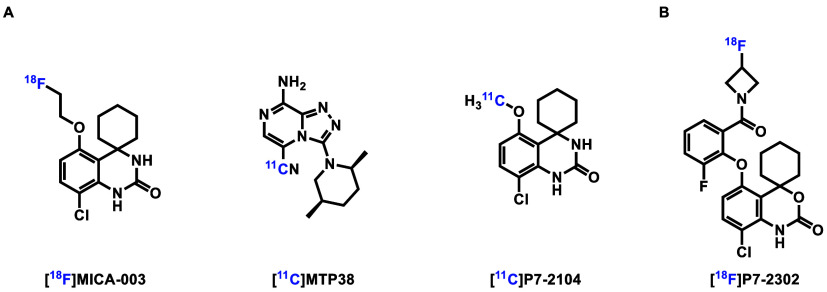
PDE7 PET ligands. (A) Previously reported PDE7 PET ligands; (B) PDE7 PET ligand developed in this study.

Positron emission tomography (PET) is a widely employed imaging technique in disease diagnosis and drug development.^17−19^ Owing to its high sensitivity, inherent quantitative performance, and deep tissue penetration, PET has become an invaluable tool for preclinical and clinical research, particularly for target quantification, receptor occupancy, and therapeutic response monitoring. Nonetheless, only a handful of PDE7 PET ligands have been developed to date,^20^ including [^18^F]MICA-003,^21^ [^11^C]MTP38,^22^ and [^11^C]P7–2104^23^ (Figure 1). [^18^F]MICA-003 had a high PDE7 inhibitory potency (IC_50_ = 17 nM) and was able to cross the blood-brain barrier (BBB); however, its utility was hampered by the rapid metabolism to 2-[^18^F]fluoroethanol *in vivo*. Recently, [^11^C]MTP38 and [^11^C]P7–2104 were reported as PDE7 PET ligands and they readily crossed the BBB; however, both probes showed only moderate *in vivo* binding specificity. Consequently, the development of suitable PDE7-targeted PET ligands remains an unmet clinical need. In this study, we designed and synthesized a series of PDE7 inhibitor candidates and developed [^18^F]**7** ([^18^F]P7–2302**)** as a PDE7 PET ligand candidate, which was further evaluated in autoradiography and PET imaging studies, as well as whole-body biodistribution and radiometabolite analysis in rodents.

## Experimental Section

### General Information

The NMR spectra were recorded on a 600 MHz spectrometer. High-resolution mass (HRMS) data were collected on a high-resolution mass spectrometer in the ESI mode. No promiscuity was observed in the assay of PAINS (Pan Assay Interference Compounds) for all candidate compounds **1**–**7**. Animal studies were performed following institutional ethical guidelines of the Institutional Animal Care and Use Committee (IACUC) of Emory University. Rodents were fed under the 12 h light/12 h dark cycle.

### Chemistry

#### Synthesis of 2-((8-Chloro-2-oxo-1,2-dihydrospiro[benzo[*d*][1,3]oxazine-4,1′-cyclohexan]-5-yl)oxy)-3-fluoro-*N*,*N*-dimethylbenzamide (**1**)

To a solution of 2-((8-chloro-2-oxo-1,2-dihydrospiro[benzo[*d*][1,3]oxazine-4,1′-cyclohexan]-5-yl)oxy)-3-fluorobenzoic acid (50 mg, 0.123 mmol, 1.0 equiv) in dry THF (2 mL), DIPEA (*N*,*N*-diisopropylethylamine, 31 mg, 0.24 mmol, 2.0 equiv) and HATU (hexafluorophosphate azabenzotriazole tetramethyl uranium, 70 mg, 0.185 mmol, 1.5 equiv) were added. The mixture was stirred at room temperature for 5 min and then Me_2_NH (40% in water, 20 mg, 0.185 mmol, 1.5 equiv) was added. The resulting mixture was stirred at room temperature for 12 h. Then the mixture was concentrated and purified by Prep-TLC and reversed-phase silica gel column chromatography (10–75% MeOH in H_2_O). And compound **1** was generated (27.2 mg, 51%) as a white solid. ^1^H NMR (400 MHz, Chloroform-*d*) δ 7.36 (s, 1H), 7.34–7.29 (m, 1H), 7.23 (ddd, *J* = 12.0, 8.4, 1.6 Hz, 1H), 7.17 (dt, *J* = 6.8, 1.2 Hz, 1H), 7.11 (d, *J* = 8.8 Hz, 1H), 6.29 (dd, *J* = 9.2, 1.6 Hz, 1H), 3.05 (s, 3H), 2.92 (s, 3H), 2.40 (s, 2H), 2.14 (d, *J* = 13.2 Hz, 2H), 1.98 (qt, *J* = 13.2, 3.6 Hz, 2H), 1.80 (d, *J* = 13.2 Hz, 1H), 1.62 (s, *J* = 13.6 Hz, 2H), 1.27 (d, *J* = 12.4 Hz, 1H). LRMS (ESI): C_22_H_23_ClFN_2_O_4_^+^ (M + H^+^): 433.1, found: 433.5.

#### Synthesis of 2-((8-Chloro-2-oxo-1,2-dihydrospiro[benzo[*d*][1,3]oxazine-4,1′-cyclohexan]-5-yl)oxy)-4-fluoro-*N*,*N*-dimethylbenzamide (**2**)

To a solution of 2-((8-chloro-2-oxo-1,2-dihydrospiro[benzo[*d*][1,3]oxazine-4,1′-cyclohexan]-5-yl)oxy)-4-fluorobenzoic acid (100.0 mg, 0.24 mmol, 1.0 equiv) in DMF (8.0 mL) was added dimethylamine (16.2 mg, 0.36 mmol, 1.5 equiv), HATU (136.0 mg, 0.36 mmol, 1.5 equiv), and DIPEA (62.0 mg, 0.48 mmol, 2.0 equiv). The mixture was stirred at room temperature for 12 h and then quenched with water. The mixture was extracted three times with ethyl acetate. The combined organic layer was washed with brine, dried over Na_2_SO_4_, filtered, and concentrated *in vacuo* to give the crude product which was purified by reverse-phase silica gel column chromatography with MeOH/H_2_O (v/v = 2:1) to give **2** (14 mg, 13% yield) as a white solid. ^1^HNMR (400 MHz, Chloroform-*d*) δ 7.42 (s, 1H), 7.36 (dd, *J* = 8.4, 6.0 Hz, 1H), 7.23 (d, *J* = 8.8 Hz, 1H), 6.94 (td, *J* = 8.0, 2.4 Hz, 1H), 6.62 (dd, *J* = 9.6, 2.4 Hz, 1H), 6.53 (d, *J* = 8.8 Hz, 1H), 3.09 (s, 3H), 2.95 (s, 3H), 2.17 (d, *J* = 14.0 Hz, 2H), 2.08 (d, *J* = 13.6 Hz, 2H), 1.98–1.88 (m, 2H), 1.77 (d, *J* = 13.6 Hz, 1H), 1.60 (s, *J* = 17.2 Hz, 2H), 1.27–1.23 (m, 1H). LRMS (ESI): C_22_H_23_ClFN_2_O_4_^+^ (M + H^+^): 433.1, found: 433.1.

#### Synthesis of 2-((8-Chloro-2-oxo-1,2-dihydrospiro[benzo[*d*][1,3]oxazine-4,1′-cyclohexan]-5-yl)oxy)-5-fluoro-*N*,*N*-dimethylbenzamide (**3**)

A solution of 2-((8-chloro-2-oxo-1,2-dihydrospiro[benzo[*d*][1,3]oxazine-4,1′-cyclohexan]-5-yl)oxy)-5-fluorobenzoic acid (140.0 mg, 0.34 mmol, 1.0 equiv) and HATU (194.0 mg, 0.51 mmol, 1.5 equiv) in CH_2_Cl_2_ (2 mL) was stirred at room temperature for 10 min. Then DIPEA (88 mg, 0.68 mmol, 2.0 equiv) and dimethylamine (18.5 mg, 0.41 mmol, 1.2 equiv) were added. The solution was stirred at room temperature for 12 h. Then the solution was quenched with water and extracted with ethyl acetate. The organic phase was washed with brine, dried over Na_2_SO_4_, and concentrated in a vacuum. The residue was purified by prep-TLC (DCM/MeOH = 20/1) to give **3** (30 mg, 20% yield) as a white solid. ^1^H NMR (400 MHz, Chloroform-*d*) δ 7.39 (s, 1H), 7.16 (d, *J* = 8.8 Hz, 1H), 7.09 (m, 2H), 6.91 (dd, *J* = 9.6, 4.0 Hz, 1H), 6.42 (d, *J* = 9.6 Hz, 1H), 3.06 (s, 3H), 2.94 (s, 3H), 2.25 (d, *J* = 8.0 Hz, 2H), 2.09 (d, *J* = 13.6 Hz, 2H), 1.99–1.89 (m, 2H), 1.78 (d, *J* = 13.2 Hz, 1H), 1.60 (d, *J* = 18.8 Hz, 2H), 1.25 (m, 1H). LRMS (ESI): C_22_H_23_ClFN_2_O_4_^+^ (M + H^+^): 433.1, found: 433.2.

#### Synthesis of 2-((8-Chloro-2-oxo-1,2-dihydrospiro[benzo[*d*][1,3]oxazine-4,1′-cyclohexan]-5-yl)oxy)-6-fluoro-*N*,*N*-dimethylbenzamide (**4**)

A solution of 2-((8-chloro-2-oxo-1,2-dihydrospiro[benzo[*d*][1,3]oxazine-4,1′-cyclohexan]-5-yl)oxy)-6-fluorobenzoic acid (104.0 mg, 0.25 mmol, 1.0 equiv) and HATU (144 mg, 0.38 mmol, 1.5 equiv) in DMF (2 mL) was stirred at 0 °C for 10 min. Then DIPEA (65.0 mg, 0.50 mmol, 2.0 equiv) and dimethylamine (67.5 mg, 1.50 mmol, 6.0 equiv) were added, and the mixture was stirred at room temperature for 12 h. Then the solution was quenched with water and extracted with ethyl acetate. The organic phase was washed with brine, dried over Na_2_SO_4_, and concentrated in a vacuum. The residue was purified by prep-TLC (DCM/MeOH = 30/1) to give **4** (30 mg, 28% yield) as a white solid. ^1^H NMR (400 MHz, Chloroform-*d*) δ 7.39 (s, 1H), 7.33 (td, *J* = 10.4, 2.0 Hz, 1H), 7.20 (d, *J* = 8.8 Hz, 1H), 6.96 (td, *J* = 12.4, 3.2 Hz, 1H), 6.70 (d, *J* = 8.4 Hz, 1H), 6.53 (d, *J* = 8.8 Hz, 1H), 3.11 (s, 3H), 2.98 (s, 3H), 2.32–2.24 (m, 1H), 2.20–2.04 (m, 3H), 1.94 (m, 2H), 1.77 (d, *J* = 13.2 Hz, 1H), 1.59 (m, 2H), 1.27 (d, *J* = 8.4 Hz, 1H). LRMS (ESI): C_22_H_23_ClFN_2_O_4_^+^ (M + H^+^): 433.1, found: 433.1.

#### Synthesis of 5-(2-(Azetidine-1-carbonyl)-6-fluorophenoxy)-8-chlorospiro[benzo[*d*][1,3]oxazine-4,1′-cyclohexan]-2(1*H*)-one (**5**)

A solution of 2-((8-chloro-2-oxo-1,2-dihydrospiro[benzo[*d*][1,3]oxazine-4,1′-cyclohexan]-5-yl)oxy)-3-fluorobenzoic acid (50.0 mg, 0.123 mmol, 1.0 equiv) and PyBOP (benzotriazolyloxy-tris[pyrrolidino]-phosphonium hexafluorophosphate, 96.0 mg, 0.185 mmol, 1.5 equiv) in CH_2_Cl_2_ (2 mL) was stirred at room temperature for 15 min. Then DIPEA (31.7 mg, 0.246 mmol, 2.0 equiv) and azetidine (8.4 mg, 0.148 mmol, 1.2 equiv) were added and the mixture was stirred at 30 °C for 3 h. Then the solution was concentrated in a vacuum. The residue was purified by prep-TLC (DCM/MeOH = 20/1) to give **5** (15 mg, 27% yield) as a white solid. ^1^H NMR (400 MHz, Chloroform-*d*) δ 7.39 (s, 1H), 7.33–7.28 (m, 1H), 7.25–7.21 (m, 2H), 7.11 (d, *J* = 9.2 Hz, 1H), 6.23 (d, *J* = 9.2 Hz, 1H), 4.12 (t, *J* = 8.0 Hz, 2H), 4.10 (s, 2H), 2.48 (s, 2H), 2.31 (p, *J* = 8.0 Hz, 2H), 2.20 (d, *J* = 13.6 Hz, 2H), 2.06–1.96 (m, 2H), 1.83 (d, *J* = 13.6 Hz, 1H), 1.65 (d, *J* = 14.8 Hz, 2H), 1.33–1.28 (m, 1H). LRMS (ESI): C_23_H_23_ClFN_2_O_4_^+^ (M + H^+^): 445.1, found: 445.1.

#### Synthesis of 2-((8-Chloro-2-oxo-1,2-dihydrospiro[benzo[*d*][1,3]oxazine-4,1′-cyclohexan]-5-yl)oxy)-*N*-cyclopropyl-3-fluoro-*N*-methylbenzamide (**6**)

A solution of 2-((8-chloro-2-oxo-1,2-dihydrospiro[benzo[*d*][1,3]oxazine-4,1′-cyclohexan]-5-yl)oxy)-3-fluorobenzoic acid (50.0 mg, 0.123 mmol, 1.0 equiv) and HATU (70.3 mg, 0.185 mmol, 1.5 equiv) in CH_2_Cl_2_ (2 mL) was stirred at room temperature for 15 min. Then DIPEA (31.7 mg, 0.246 mmol, 2.0 equiv) and *N*-methylcyclopropanamine (10.5 mg, 0.148 mmol, 1.2 equiv) were added, and the mixture was stirred at room temperature for 12 h. Then the solution was concentrated in a vacuum. The residue was purified by prep-TLC (DCM/MeOH = 20/1) and then purified by prep-HPLC to give **6** (24 mg, 43% yield) as a white solid. ^1^H NMR (400 MHz, DMSO-*d*_6_) δ 9.74 (s, 1H), 7.47 (t, *J* = 8.4 Hz, 1H), 7.44–7.39 (m, 1H), 7.34 (d, *J* = 7.6 Hz, 1H), 7.28 (d, *J* = 9.2 Hz, 1H), 6.24 (d, *J* = 8.8 Hz, 1H), 2.90 (s, 3 H), 2.66 (s, 1H), 2.39–2.13 (m, 2H), 1.98 (d, *J* = 8.0 Hz, 2H), 1.79–1.72 (m, 3H), 1.58 (d, *J* = 12.8 Hz, 2H), 1.20 (m, 1H), 0.53 (s, 4H). LRMS (ESI): C_24_H_25_ClFN_2_O_4_^+^ (M + H^+^): 459.1, found: 459.2.

#### Synthesis of 8-Chloro-5-(2-fluoro-6-(3-fluoroazetidine-1-carbonyl)phenoxy)spiro[benzo[*d*][1,3]oxazine-4,1′-cyclohexan]-2(1*H*)-one (**7**)

To a solution of 2-((8-chloro-2-oxo-1,2-dihydrospiro[benzo[*d*][1,3]oxazine-4,1′-cyclohexan]-5-yl)oxy)-3-fluorobenzoic acid **8a** (100 mg, 0.25 mmol, 1 equiv) and HATU (hexafluorophosphate azabenzotriazole tetramethyl uronium, 141 mg, 0.37 mmol, 1.5 equiv) in DMF (10 mL), 3-fluoroazetidine (38 mg, 0.5 mmol, 2 equiv) and DIPEA (*N*,*N*-diisopropylethylamine, 95 mg, 0.74 mmol, 3 equiv) were added, and stirred at room temperature for 12 h. Then the solvent was removed, and the residue was purified by Prep-TLC to give **7** (85 mg, 74%) as a white solid. ^1^H NMR (400 MHz, Chloroform-*d*): δ 7.40 (s, 1H), 7.35–7.28 (m, 2H), 7.24 (d, *J* = 7.3, 1H), 7.11 (d, *J* = 8.9 Hz, 1H), 6.21 (d, *J* = 8.9 Hz, 1H), 5.32 (dm, *J* = 56.2 Hz, 1H), 4.39–4.16 (m, 4H), 2.41 (m, 2H), 2.20 (d, *J* = 13.3 Hz, 2H), 1.99 (m, 2H), 1.81 (d, *J* = 12.3 Hz, 1H), 1.65–1.61 (m, 2H), 1.31–1.26 (m, 1H). ^13^C NMR (150 MHz, Chloroform-*d*): δ 166.5, 154.7 (d, *J* = 253.9 Hz), 152.9, 149.5, 138.2 (d, *J* = 13.2 Hz), 132.5, 131.0, 129.2, 127.3 (d, *J* = 8.0 Hz), 123.9, 119.3 (d, *J* = 18.8 Hz), 115.4, 113.1, 109.7, 85.7, 81.7 (d, *J* = 203.8 Hz), 58.9 (d, *J* = 26.9 Hz), 56.4 (d, *J* = 25.3 Hz), 39.8, 25.0, 20.9, 20.8. LRMS (ESI): C_23_H_22_ClF_2_N_2_O_4_^+^ (M + H^+^): 463.1, found: 463.1. HRMS (ESI): exact mass calcd for C_23_H_22_ClF_2_N_2_O_4_^+^ (M + H^+^): 463.1231, found: 463.12305.

#### Synthesis of 8-Chloro-5-(2-fluoro-6-(3-hydroxyazetidine-1-carbonyl)phenoxy)spiro[benzo[*d*][1,3]oxazine-4,1′-cyclohexan]-2(1*H*)-one (**9**)

To a solution of 2-((8-chloro-2-oxo-1,2-dihydrospiro[benzo[*d*][1,3]oxazine-4,1′-cyclohexan]-5-yl)oxy)-3-fluorobenzoic acid **8a** (80 mg, 0.2 mmol, 1 equiv) in DMF (8 mL), HATU (hexafluorophosphate azabenzotriazole tetramethyl uronium, 91 mg, 0.24 mmol, 1.2 equiv) and DIPEA (*N*,*N*-diisopropylethylamine, 31 mg, 0.24 mmol, 1.2 equiv) were added at 0 °C. The mixture was stirred at 0 °C for 30 min, then azetidin-3-olhydrochloride (22 mg, 0.2 mmol, 1 equiv) was added, and stirred at room temperature for 6 h. Then water was added, and the mixture was extracted with EtOAc. The organic phase was dried over Na_2_SO_4_, and concentrated to give **9** (crude, yellow oil).

#### Synthesis of 1-(2-((8-Chloro-2-oxo-1,2-dihydrospiro[benzo[*d*][1,3]oxazine-4,1′-cyclohexan]-5-yl)oxy)-3-fluorobenzoyl)azetidin-3-yl 4-methylbenzenesulfonate (**10**)

To a solution of **9** (108 mg, 0.23 mmol, 1 equiv) in CH_2_Cl_2_ (8 mL), Et_3_N (46 mg, 0.46 mmol, 2 equiv), DMAP (4-dimethylaminopyridine, 5.6 mg, 0.046 mmol, 0.2 equiv), and TsCl (4-toluenesulfonyl chloride, 65 mg, 0.34 mmol, 1.5 equiv) were added at room temperature and stirred for 12 h. The solvent was removed, and the residue was purified by column chromatography to give **10** (53 mg, white solid, 38% yield over two steps). ^1^H NMR (400 MHz, Chloroform-*d*): δ 7.77 (d, *J* = 8.5, 2H), 7.38–7.36 (m, 3H), 7.32–7.27 (m, 2H), 7.21 (d, *J* = 6.7, 1H), 7.10 (d, *J* = 8.8 Hz, 1H), 6.17 (d, *J* = 9.2 Hz, 1H), 5.04 (m, 1H), 4.27–3.98 (m, 4H), 2.47 (s, 3H), 2.38 (m, 2H), 2.16 (d, *J* = 12.8 Hz, 2H), 1.96 (m, 2H), 1.79 (d, *J* = 12.1 Hz, 1H), 1.66–1.64 (m, 2H), 1.30–1.25 (m, 1H). ^13^C NMR (150 MHz, Chloroform-*d*): δ 166.2 (d, *J* = 3.3 Hz), 154.9 (d, *J* = 254.4 Hz), 152.8, 149.5, 146.1, 138.2 (d, *J* = 12.5 Hz), 132.6, 132.5, 130.4, 129.2, 128.0, 127.3 (d, *J* = 6.7 Hz), 124.0, 119.5 (d, *J* = 17.7 Hz), 113.1, 109.6, 85.7, 67.0, 58.5, 55.5, 25.0, 21.9, 20.9, 20.8. LRMS (ESI): C_30_H_29_ClFN_2_O_7_S^+^ (M + H^+^): 615.1, found: 615.1. HRMS (ESI): calculated for C_30_H_29_ClFN_2_O_7_S^+^ (M+H^+^): 615.1363; found: 615.13642.

### Phosphodiesterase Inhibition Assay

The inhibition assay of phosphodiesterase 7 with compounds **1**–**7** was performed by Eurofins. The inhibition assay of other phosphodiesterases with compound **7** was performed by Reaction Biology Corporation at a concentration of 3 μM.

### Molecular Docking

Since the structure of PDE7B is undetermined, a structural model was predicted with the AlphaFold protocol implemented in ColabFold. The query sequence was obtained from UniProt (ID: Q9NP56 PDE7B_Human) for multiple sequence alignment (mmseqs2_uniref_env). Predictions were performed without templates with 6 recycles per model. The model with the highest reported confidence (pLDDT = 78.7 and a pTM = 0.729) was selected for energy minimization with Amber ff14SB and used in the subsequent study. Docking studies were performed with AutoDock Vina (version 1.2.5) and visualized in ChimeraX (version 1.7.1). 2D interaction plots were generated using Schrödinger Maestro (version 13.8.135).

### Radiochemistry

[^18^F]Fluoride (30 mCi, 1110 MBq) in ^18^O-water was dried with tetraethylammonium bicarbonate (1 mg/0.5 mL in MeOH) in a vial at 110 °C with N_2_. When there was no liquid left, anhydrous MeCN (1 mL) was added and dried again, and this drying process was repeated twice. Then tosylate precursor **10** (1 mg) in DMF/*t*BuOH (100/300 μL) was added to dried [^18^F]Et_4_NF and heated at 140 °C for 10 min. [^18^F]**7** was purified by HPLC with a Phenomenex Luna 5 μm C18(2) 100 Å Prep column (10 mm × 250 mm) and MeCN/H_2_O (v/v = 45/55, containing 0.1% NEt_3_, 5 mL/min) as eluting buffer. [^18^F]**7** was obtained in 10% RCY (decay corrected) with molar activity of 152 GBq/μmol.

### *In Vitro* Autoradiography in Rat Brains

Autoradiography studies were conducted as previously reported.^24,25^ In brief, SD rat brain sections (20 μM) were preincubated with buffer (Tris-HCl, 50 mM) at ambient temperature for 20 min and then incubated with [^18^F]**7** (1 μCi/mL) for 30 min. After that, the brain sections were washed with cold buffer and water, dried with cold air, and exposed to the phosphor plate (12 h). In blocking studies, brain sections were incubated with [^18^F]**7** in the presence of compound **7** (10 μM) or P7–2104 (10 μM).

### PET Imaging in Rat Brains

PET imaging was carried out as previously reported.^26^ [^18^F]**7** (30–40 uCi) was administered to SD rats via the tail vein, and dynamic scans were conducted with a G8 scanner (Sofie) for 1 h (*n* = 4). In blocking studies, elacridar (5 mg/kg) was administered intravenously at 20 min before the administration of [^18^F]**7**, and P7–2104 or **7** (3 mg/kg) was administered intravenously at 10 min before the administration of [^18^F]**7** (*n* = 3). PET data were analyzed with PMOD (PMOD Technologies LLC, Switzerland).

### *Ex Vivo* Whole-Body Biodistribution in CD-1 Mice

Whole-Body biodistribution was carried out as previously reported.^27,28^ [^18^F]**7** (10 μCi/100 μL) was administered intravenously to CD-1 mice, which were sacrificed at 5, 15, 30, and 60 min after administration of [^18^F]**7** (*n* = 3). Tissues of interest were collected, weighted, and measured by a γ counter.

### Radiometabolic Analysis in Rats

[^18^F]**7** was injected via the tail vein of SD rats and then euthanized 30 min after administration of [^18^F]**7** (0.1 mCi per rat, *n* = 2). The brain was collected, homogenized, and cold acetonitrile (0.2 mL) was added then centrifuged (14 000*g*, 5 min) at 4 °C. This step was repeated until there was no obvious precipitation formed. Then the supernatant was injected into a radioHPLC with unlabeled **7** as the internal standard. Both [^18^F]**7** and its ^18^F-metabolites were collected by HPLC and then measured by γ counter. The same procedure was conducted for plasma.

### Measurement of Log *D*

PBS buffer (0.01 M) and *n*-octanol were presaturated with each other overnight before the measurement. The PDE7 ligand [^18^F]**7**, PBS (0.01 M, 5 mL), and *n*-octanol (5 mL) were combined and vortexed for 3 min, and then centrifuged (∼14 000 rpm, 5 min). The PBS fraction and *n*-octanol fraction were weighted and counted by γ counter. The log* D* was calculated as log[(radioactivity_*n*-octanol_/weight_*n*-octanol_)/(radioactivity_PBS_/weight_PBS_)].

## Results and Discussion

### Chemistry

**Scheme 1:**
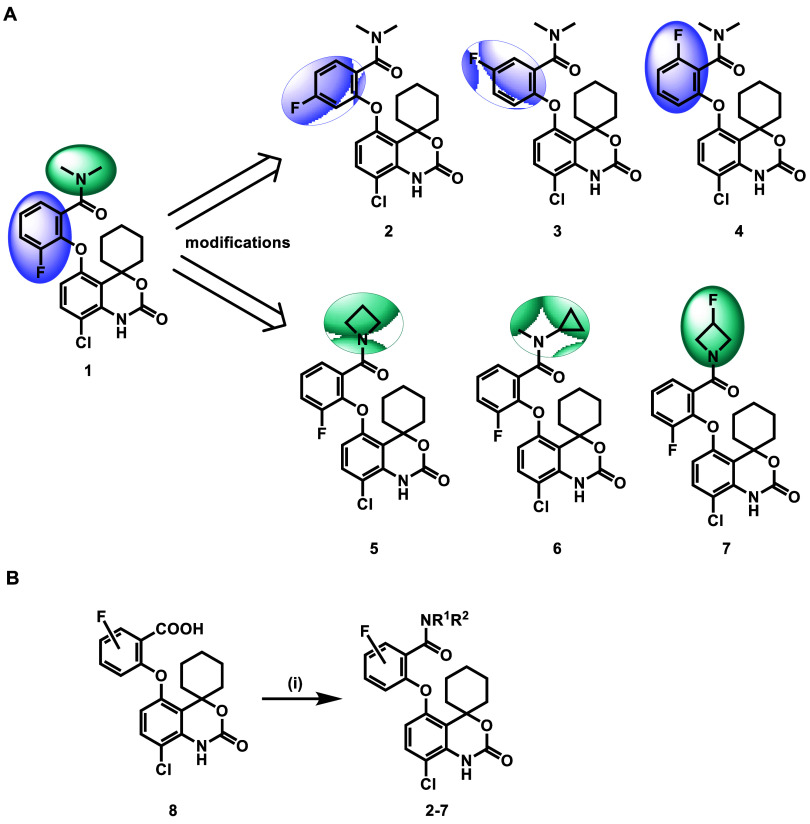
Design and Synthesis of PDE7 Inhibitor Candidates (A) Design of PDE7 inhibitor candidates suitable for radiofluorination; (B) synthesis of PDE7 inhibitor candidates **1–7**. (i) amines, HATU or PyBOP, DIPEA, DMF or CH_2_Cl_2_, room temperature, 3–12 h, 13–74% yields. HATU = hexafluorophosphate azabenzotriazole tetramethyl uranium, PyBOP = benzotriazolyloxy-tris[pyrrolidino]-phosphonium hexafluorophosphate, DIPEA = *N*,*N*-diisopropylethylamine, DMF = dimethylformamide.

To develop potent and selective PDE7 inhibitors for ^18^F-fluorination and PET imaging, we designed a series of PDE7 inhibitor candidates based on compound **1**, which exhibited a high binding affinity (*K*_D_ = 0.8 nM) toward PDE7 and reasonable brain uptake (0.25%ID/g).^29^ Modifications on the aryl and amide moieties led to new PDE7 inhibitor candidates **2**–**7**. As shown in Scheme 1, compounds **1**–**7** were synthesized via the condensation of acids **8** and respective amines in 13–74% yields in the presence of hexafluorophosphate azabenzotriazole tetramethyl uranium (HATU) or benzotriazolyloxy-tris[pyrrolidino]-phosphonium hexafluorophosphate (PyBOP).

### Pharmacology

**Figure 2:**
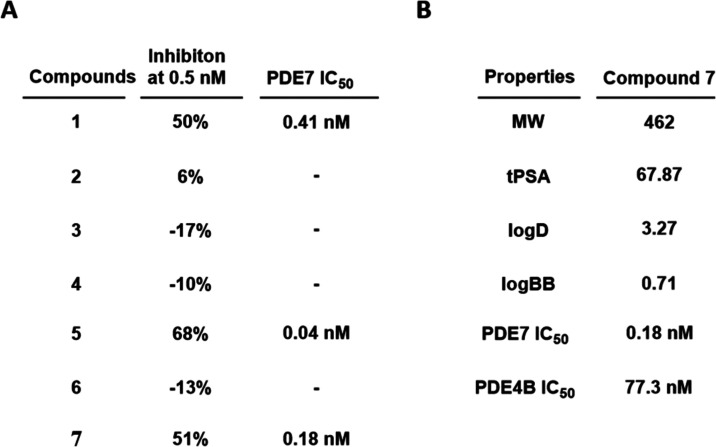
Pharmacological profiles of compounds **1**–**7**. (A) Inhibition assay of PDE7 with compounds **1**–**7**; (B) representative pharmacological and physicochemical properties of compound **7**.

To investigate the potency of compounds **1**–**7** toward PDE7, we evaluated PDE7%inhibition with compounds **1**–**7** at a fixed concentration (Figure 2A). The modification of fluorine positions on the aryl group (compounds **2**–**4**) did not improve potency. Replacing the *N*,*N*-dimethylamino group with azetidin-1-yl (compound **5**) enhanced the inhibitory potency, while the incorporation of *N*-cyclopropyl-*N*-methylamino group (compound **6**) was less effective. To further introduce a fluorine atom suitable for radiofluorination, the fluorine atom was incorporated into the azetidin-1-yl group (compound **7**), which preserved high potency (IC_50_ = 0.18 nM**)**. We further investigated the selectivity of compound **7** toward PDE7 over other PDEs (Figures 2B and S1). In the phosphodiesterase inhibition assays with other PDEs at the concentration of 3 μM, compound **7** was moderately active against PDE4, while no activity was detected against other PDEs subfamilies (<50% inhibition). Among four PDE4 subtypes, compound **7** had the highest potency toward PDE4B (92% inhibition), and the concentration-inhibition relationship of compound **7** toward PDE4B was further determined (IC_50_ = 77.3 nM). Overall, compound **7** had a favorable target selectivity profile, with >400-fold selectivity for PDE7 over other PDE subfamilies. The topological polar surface area (tPSA = 67.87) of compound **7** was predicted by ChemDraw and the lipophilicity (log *D* = 3.27) of compound **7** was determined by the shake flask method,^30^ suggesting appropriate physicochemical properties of compound **7**. The BBB permeability of compound **7** was also predicted by ACD/Percepta, and the results (log BB = 0.71) indicated a high possibility (log BB > −1) of BBB permeability. In addition, we investigated the off-target binding profile of compound **7** against 66 major CNS targets, including transporters, ion channels, and GPCR enzymes (Figure S2). No significant off-target binding (>50% inhibition) was observed for compound **7** at 10 μM, except the 5-HT2B (*K*_i_ = 3364 nM) and Sigma1 (*K*_i_ = 7408 nM).

### Molecular Docking

**Figure 3:**
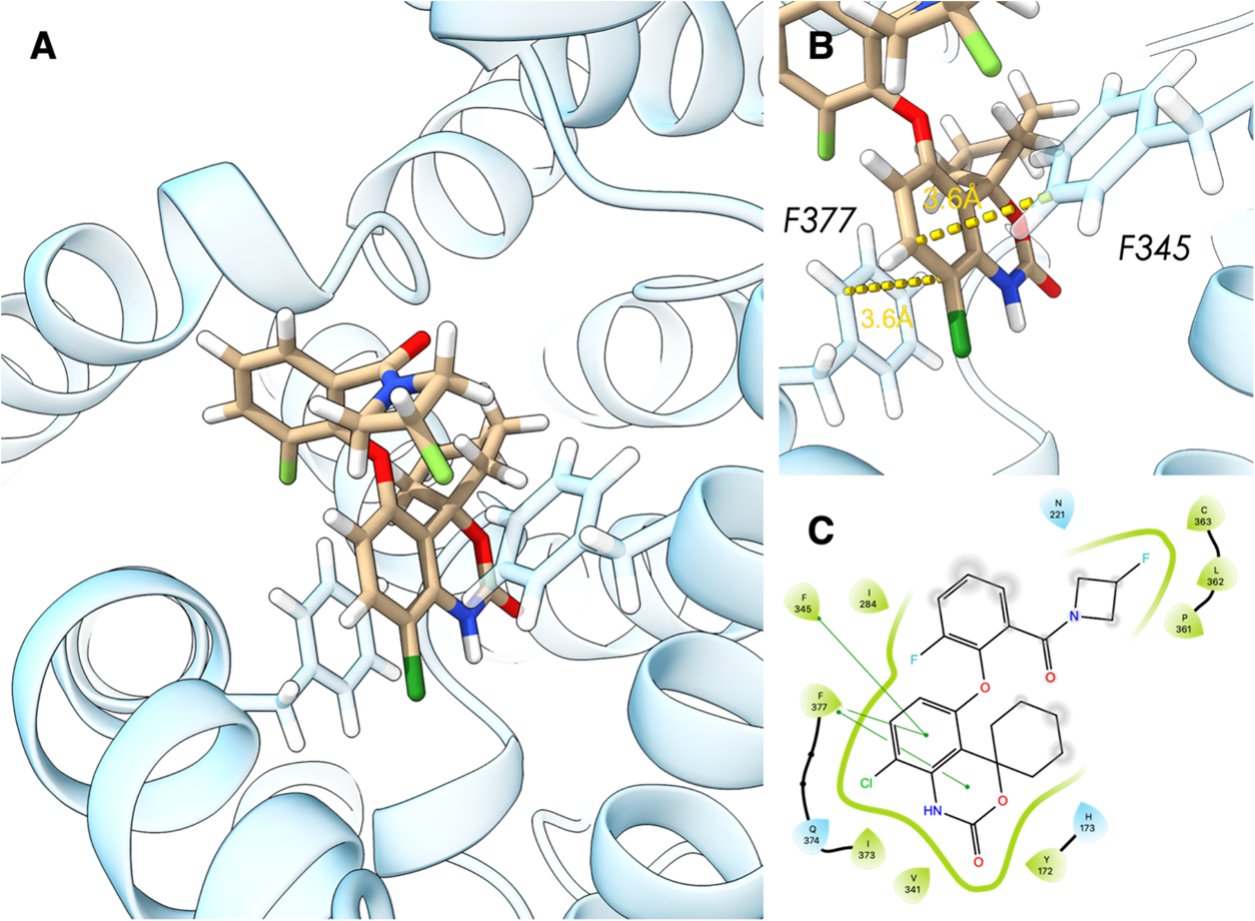
Molecular docking structure of compound **7** onto PDE7. (A) Binding pose of compound **7** (tan) with PDE7B (blue); (B) close-up view of π–π interactions with residues F345 and F377; (C) the 2D interaction plot shows the binding pocket composed of mainly hydrophobic interactions (light green) with π–π interactions shown as dark green lines.

Computational docking studies were conducted to investigate possible interactions between compound **7** and PDE7B. The analysis showed that the ligand predominantly interacts with a hydrophobic binding pocket of PDE7B, which is made up of residues I284, P361, L362, V341, F345, I373, and F377 (Figure 3). In particular, compound **7** is stabilized in the pocket by favorable π–π interactions with residues F345 and F377. The interactions observed with residue F345 were parallel-displaced π–π stacking, whereas, for F377, the interaction was T-shape stacking, both at a distance of around 3.6 Å. These results suggested that compound **7** exhibited favorable interactions with PDE7B.

### Radiochemistry

**Scheme 2:**
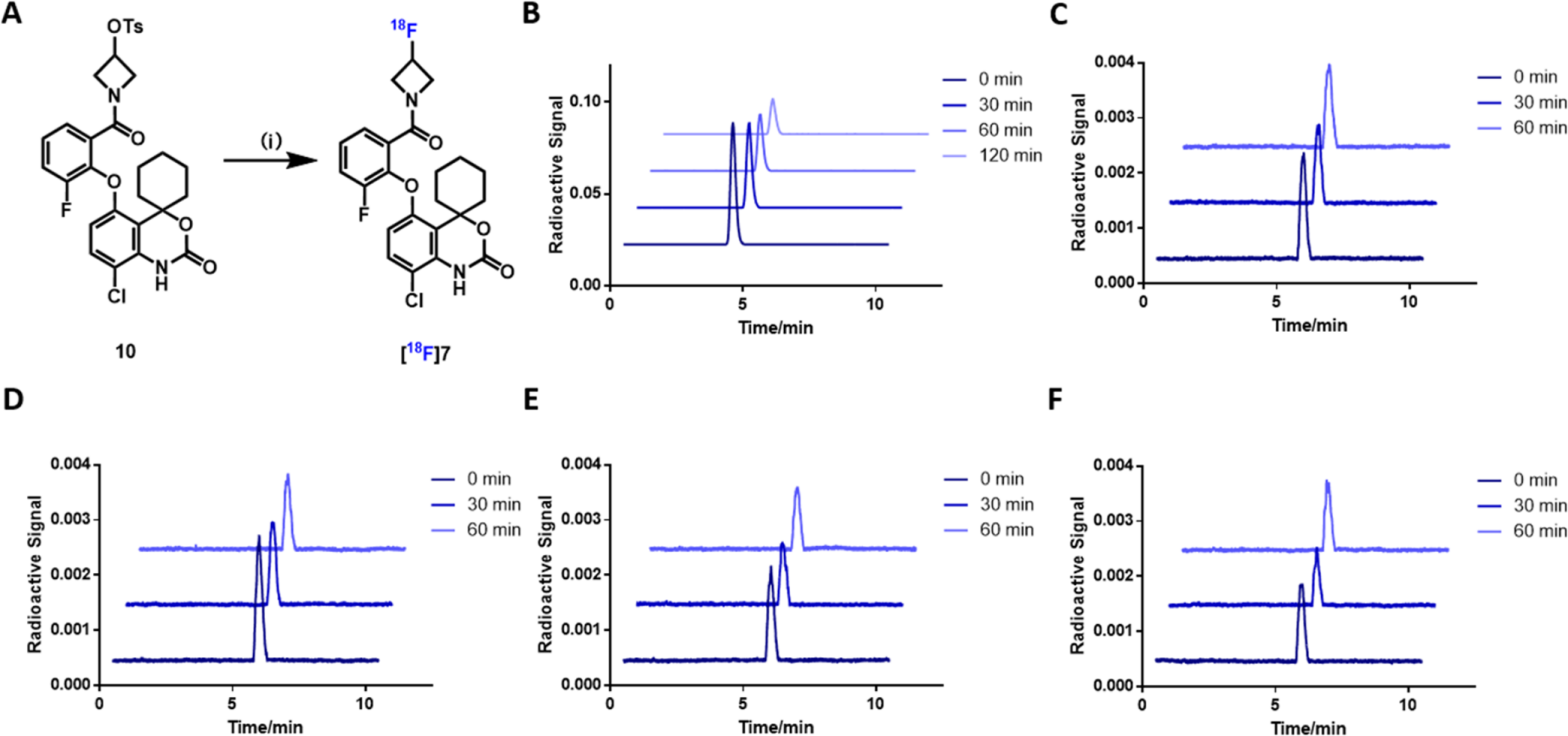
Radiosynthesis of [^18^F]**7** and Tracer Stability Tests (A) Radiosynthesis of [^18^F]**7**, conditions: (i) [^18^F]Et_4_NF, DMF/*t*BuOH (v:v = 1:3), 140 °C, 10 min, 10% RCY; (B) stability of [^18^F]**7** in saline; (C–F) stability of [^18^F]**7** in mouse, rat, NHP, and human serums. DMF = dimethylformamide.

Given compound **7**’s favorable pharmacological and physicochemical properties, it was labeled with fluorine-18 and further evaluated as a PDE7 PET ligand.^18,31^ The tosylate precursor **10** for ^18^F-labeling was synthesized in two steps (Scheme S1). The condensation of acid **8a** and azetidin-3-ol provided key intermediate **9**, followed by conversion of the hydroxyl group with 4-toluenesulfonyl chloride under basic conditions to afford tosylate precursor **10** in 38% yield over two steps. As shown in Scheme 2, the radiosynthesis of [^18^F]**7** was conducted via nucleophilic substitution reaction of the corresponding tosylate precursor **10** with [^18^F]Et_4_NF in a solvent mixture of DMF and *t*BuOH (v:v = 1:3) at 140 °C for 10 min. [^18^F]**7** was generated in 10% radiochemical yield (RCY, decay-corrected) with a molar activity of 152 GBq/μmol. The radiochemical purity of [^18^F]**7** was greater than 99%. Additionally, to have an insight of *in vitr*o stability of [^18^F]**7**, we performed stability tests in formulation solution and serums from different species. The excellent *in vitro* stability of [^18^F]**7** was confirmed, and no obvious decomposition or metabolite was observed in saline, mouse, rat, NHP, and human serums for up to 60 or 120 min.

### *In Vitro* Autoradiography

**Figure 4:**
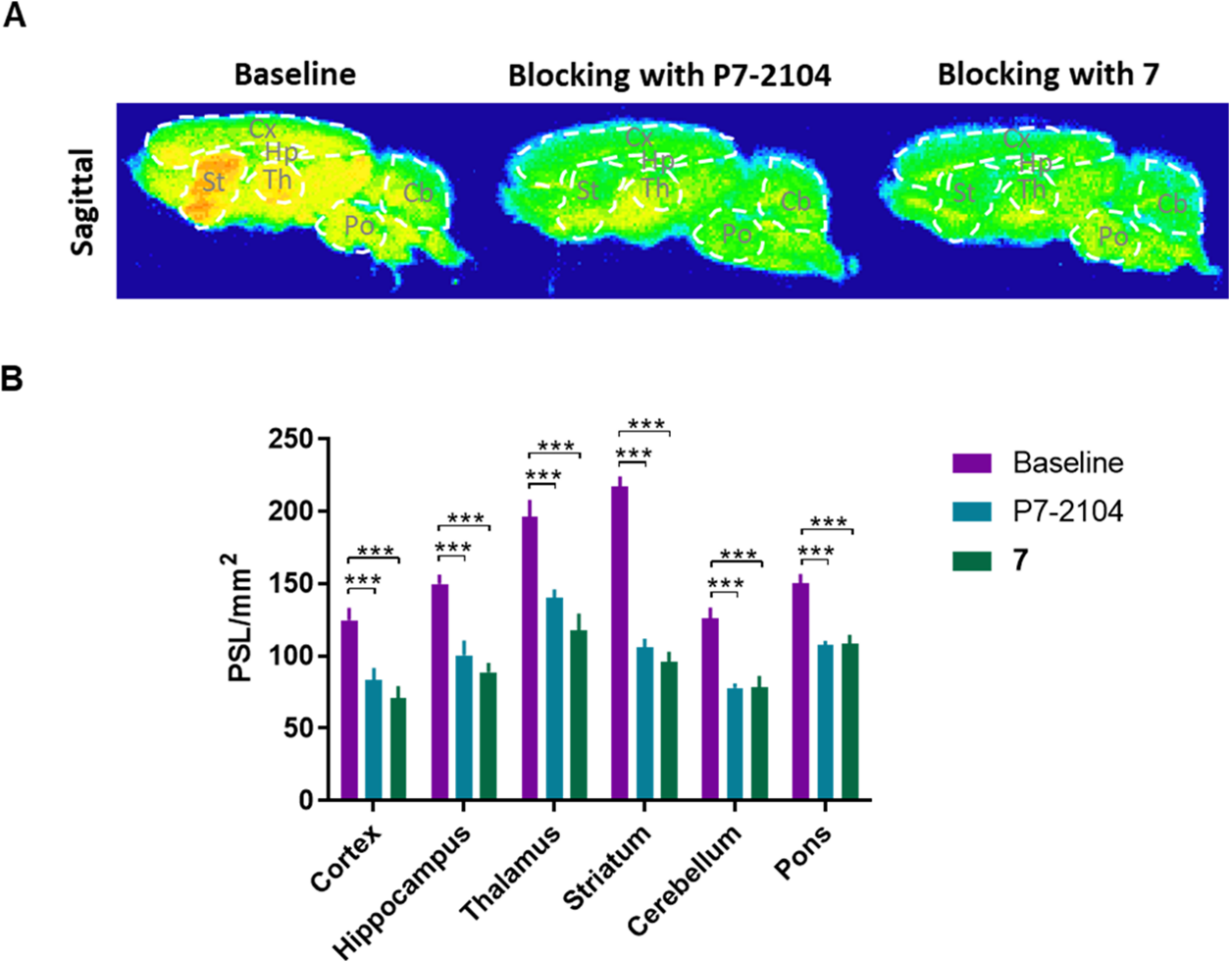
*In vitro* autoradiography studies with [^18^F]**7** in rat brains. (A) Representative images of autoradiography studies with [^18^F]**7** in baseline and blocking (P7–2104 or **7**, 10 μM) conditions on sagittal rat brain sections; (B) quantification of *in vitro* autoradiography studies with [^18^F]**7**. Cx, cortex; Hp, hippocampus; Th, thalamus; St, striatum; Cb, cerebellum; Po, pons. All data are mean ± SD, *n* ≥ 6. Statistical analysis was calculated by one-way analysis of variance (ANOVA) test. (*** *P* ≤ 0.001).

With [^18^F]**7** in hand, we carried out *in vitro* autoradiography studies on rat brain sections to validate *in vitro* binding specificity of [^18^F]**7** toward PDE7 (Figure 4). In baseline studies, [^18^F]**7** demonstrated a heterogeneous brain distribution, and high radioactivity accumulation was observed in the striatum, followed by the thalamus, cortex, hippocampus, and lowest in the pons, which is consistent with the expression of PDE7.^29,32^ In blocking studies with PDE7 inhibitors P7–2104 or **7** (10 μM), a significant reduction in radioactivity signal was observed in all brain regions, which was most pronounced in PDE7-rich regions. For example, the striatum showed 51% and 56% reductions in the blocking studies with inhibitors P7–2104 and **7**, respectively, and the heterogeneous distribution pattern of [^18^F]**7** vanished under blocking conditions. These results indicated the high *in vitro* binding specificity of [^18^F]**7** toward PDE7.

### PET Imaging

**Figure 5:**
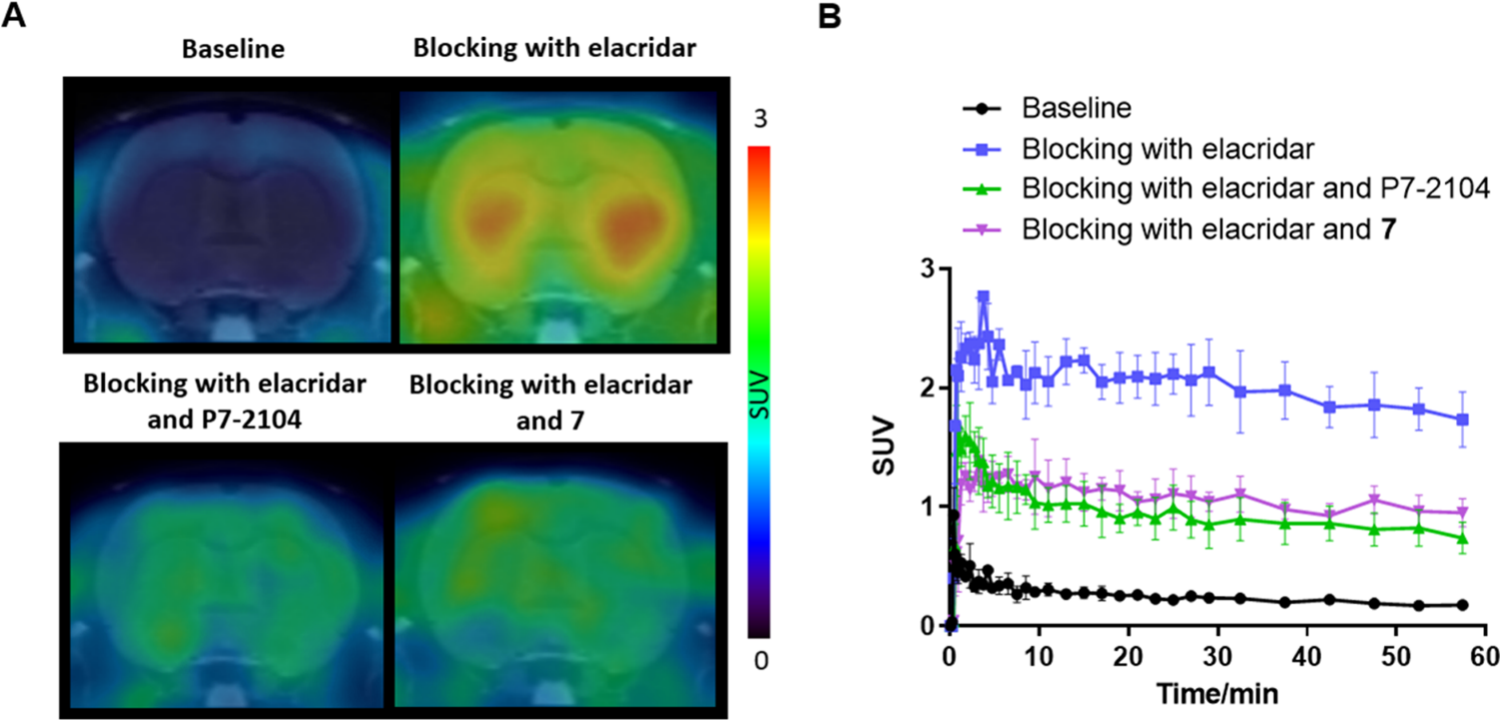
PET imaging studies of [^18^F]**7** in rat brains. (A) Representative summed (0–60 min) PET images with [^18^F]**7** in baseline and blocking (elacridar, elacridar and P7–2104, or elacridar and **7**; elacridar in 5 mg/kg, P7–2104 and **7** in 3 mg/kg) conditions; (B) TACs of [^18^F]**7** in the striatum; all data are mean ± SEM, *n* ≥ 3.

Encouraged by the positive results of *in vitro* autoradiography studies of [^18^F]**7**, we further performed PET imaging experiments in Sprague–Dawley (SD) rats to evaluate the *in vivo* binding specificity of [^18^F]**7** toward PDE7 (Figure 5). In baseline studies, [^18^F]**7** did not effectively cross the BBB with low uptake across all brain regions, including the striatum (SUV_peak_ = 0.35). To confirm whether [^18^F]**7** was a substrate of P-glycoprotein (P-gp) and/or breast cancer resistance protein (BCRP), we conducted PET imaging studies in rats pretreated with elacridar, a combined P-gp and BCRP inhibitor. The rats were pretreated with elacridar (5 mg/kg) intravenously at 20 min prior to the administration of [^18^F]**7**. Remarkably, the blockade of these efflux transporters enhanced brain uptake of [^18^F]**7**, particularly in the PDE7-rich striatum (SUV_peak_ increased by 700%, from 0.35 to 2.8). Subsequently, we sought to assess the *in vivo* binding specificity of [^18^F]**7** toward PDE7. As such, rats were pretreated with elacridar (5 mg/kg) and P7–2104 (PDE7 inhibitor, 3 mg/kg) or nonradioactive compound **7** (3 mg/kg) before the administration of [^18^F]**7**, respectively. The uptake of [^18^F]**7** decreased in all brain regions, and most markedly in the PDE7-rich striatum (SUV_peak_ reduced by 58% and 51% in the blocking studies with P7–2104 and **7**, respectively). These results suggested that the low baseline uptake of [^18^F]**7** in the brain may be attributed to P-gp/BCRP efflux, and [^18^F]**7** exhibited high *in vivo* binding specificity toward PDE7 following efflux transporter inhibition.

### *Ex Vivo* Whole-Body Biodistribution

**Figure 6:**
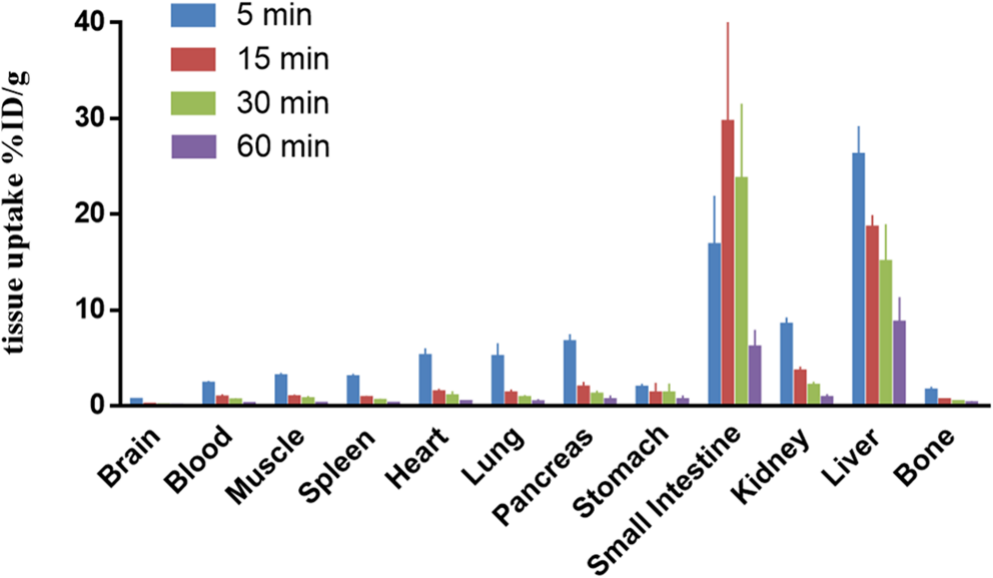
*Ex vivo* whole-body biodistribution study of [^18^F]**7** in CD-1 mice. All data are mean ± SD, *n* = 3.

To assess the whole-body distribution of [^18^F]**7**, we performed *ex vivo* whole-body biodistribution study of [^18^F]**7** in CD-1 mice. As shown in Figure 6, the radioactivity signals in major organs were measured at 5, 15, 30, and 60 min post administration of [^18^F]**7**. Initially, high radioactivity was observed in the liver, small intestine, kidney, pancreas, lung, and heart (>5%ID/g), and low uptake was found in the brain, which is consistent with the results of PET baseline imaging studies. After the initial uptake, an evident washout was observed in all major organs, except the small intestine. The high uptake and clearance in the liver, small intestine, and kidney indicated a combined hepatobiliary and urinary elimination of the parent and potential radiometabolites. The bone uptake of [^18^F]**7** was low (1.6%ID/g at 5 min) and decreased over time (0.3%ID/g at 60 min), indicating minimal radiodefluorination *in vivo*, which is consistent with PET observations.

### *Ex Vivo* Metabolite Analysis

**Figure 7:**
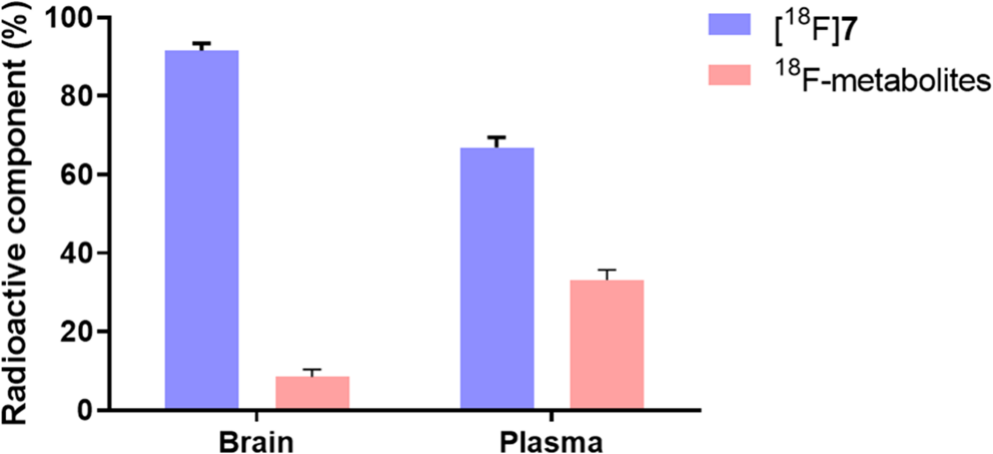
Radiometabolite analysis of [^18^F]**7** in rat brain and plasma at 30 min post injection. All data are mean ± SD, *n* = 2.

To evaluate the metabolic stability of [^18^F]**7***in vivo*, we performed radiometabolite analysis of [^18^F]**7** in rat brain and plasma following intravenous tracer administration (Figure 7). At 30 min post administration of [^18^F]**7**, the intact parent fractions were 92% and 67% in the brain and plasma, respectively. These results indicated that [^18^F]**7** exhibited suitable *in vivo* stability profile for CNS-targeted imaging.

## Conclusions

We designed and synthesized a series of PDE7 inhibitor candidates based on a known PDE7 inhibitor. In pharmacological evaluations, compounds **5** and **7** demonstrated superior inhibition of PDE7 compared to other candidates. Compound **7**, featuring a fluorinated azetidine moiety suitable for radiofluorination, was selected for further studies. Compound **7** exhibited high PDE7 potency (IC_50_ = 0.18 nM) and target selectivity (>400 folds over other PDEs). Furthermore, [^18^F]**7** (also named as [^18^F]P7–2302**)** was successfully labeled with fluorine-18, with reasonable radiochemical yield and high molar activity. Autoradiography studies showed heterogeneous distribution and excellent PDE7-specificity *in vitro*. PET imaging studies demonstrated low brain uptake due to P-gp/BCRP efflux. Inhibition of brain efflux boosted the brain uptake of [^18^F]**7**, demonstrating heterogeneous brain distribution, favorable brain kinetics, and high *in vivo* binding specificity. In summary, these findings suggest that [^18^F]**7** ([^18^F]P7–2302**)** exhibited promising performance characteristics as a PDE7-specific PET radioligand if BBB permeability is improved, which is the focus of future medicinal chemistry efforts.

## Supporting Information Available

Inhibition of other phosphodiesterases by compound **7** at 3 μM; off-target pharmacological evaluations of compound **7** against 66 major CNS targets, including common GPCRs, enzymes, ion channels, and transporters: initial screening at a concentration of 10 μM; whole-body *ex vivo* biodistribution study of [^18^F]**7** in CD-1 mice. All data are mean ± SD, *n* = 3; HPLC radio-chromatograms, and NMR spectra (PDF)
