# Supplementary material for: Development of a Novel 18F-Labeled Radioligand for Imaging Phosphodiesterase 7 with Positron Emission Tomography
Source: Mol Pharm. 2025 Feb 19;22(3):1657–66. doi: 10.1021/acs.molpharmaceut.4c01379 (PMC11881136; doi:10.1021/acs.molpharmaceut.4c01379)

Supporting Information

Development of a novel ^{18}F-labeled radioligand for imaging phosphodiesterase 7 with positron emission tomography

Jian Rong[1], Chunyu Zhao[1], Ahmad F. Chaudhary[1], Evan Jones[2], Richard Van[2], Zhendong Song[1], Yinlong Li[1], Jiahui Chen[1], Xin Zhou[1], Jimmy S. Patel[1,3], Yabiao Gao[1], Zhenkun Sun[4], Siyan Feng[1], Zachary Zhang[1], Thomas L. Collier[1], Chongzhao Ran[5], Achi Haider[1], Yihan Shao[2], Hongjie Yuan[4], and Steven H. Liang[1,*]

[1]*Department of Radiology and Imaging Sciences, Emory University, Atlanta, GA 30322, United States*

[2]*Department of Chemistry and Biochemistry, University of Oklahoma, Norman, OK 73019, United States*

[3]*Department of Radiation Oncology, Winship Cancer Institute of Emory University, Atlanta, GA 30322, United States*

[4]*Department of Pharmacology and Chemical Biology, Emory University School of Medicine, Atlanta, GA 30322, United States*

[5]*Athinoula A. Martinos Center for Biomedical Imaging, Department of Radiology, Massachusetts General Hospital and Harvard Medical School, Boston, MA 02114, United States*

*E-mail: steven.liang@emory.edu

Table of contents

1. Supporting Scheme, Figures, and Tables S3
Scheme S1 Synthesis of precursor **10** S3
Figure S1 Inhibition of other phosphodiesterases by compound **7** S3
Figure S2 Off-target pharmacological evaluation of compound **7** S4
Table S1 Whole-body biodistribution study of [^{18}F]**7** in CD-1 mice. S4

2. HPLC radio-chromatograms. S4

3. NMR spectra of isolated compounds S8

1. Supporting scheme, figures, and tables

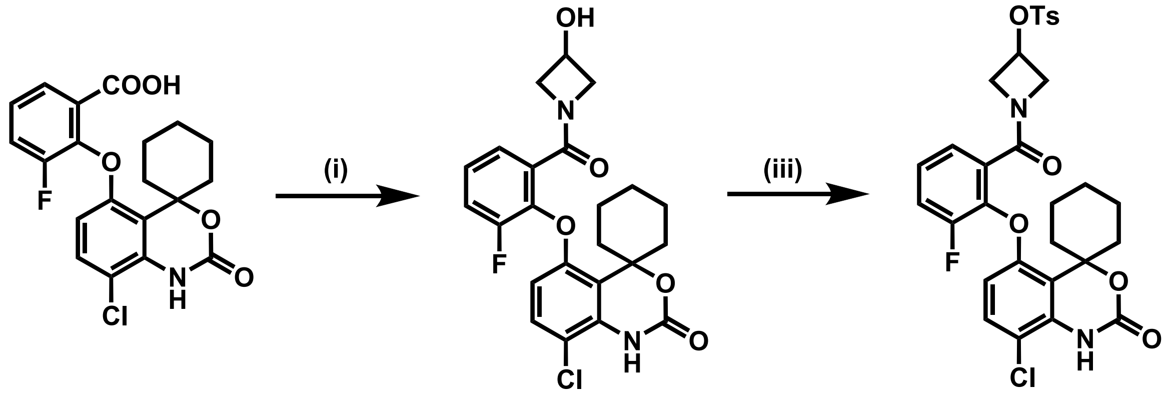

8a **9** **10**

Scheme S1. Synthesis of precursor **10**. (i) azetidin-3-ol hydrochloride, HATU, DIPEA, DMF, 0 ^{o}C, 6 h; (ii) 4-toluenesulfonyl chloride, Et_3N, 4-dimethylaminopyridine, CH_2Cl_2, room temperature, 12 h, 38% over two steps. HATU = hexafluorophosphate azabenzotriazole tetramethyl uranium, DIPEA = *N*,*N*-diisopropylethylamine, DMF = dimethylformamide.

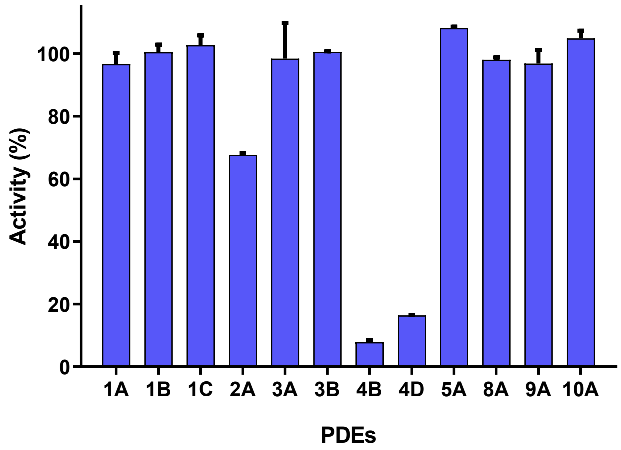

Figure S1. Inhibition of other phosphodiesterases by compound **7** at 3 μM. All data are mean ± SD, n ≥ 2.

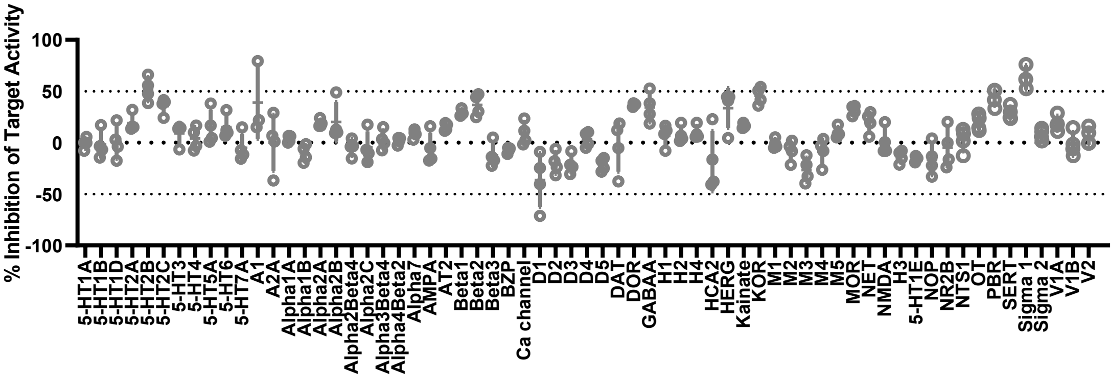

Figure S2. Off-target pharmacological evaluations of compound **7** against 66 major CNS targets, including common GPCRs, enzymes, ion channels, and transporters: initial screening at a concentration of 10 µM. All data are mean ± SD ($n \geq 3$). No significant off-target binding (> 50%) was observed, except 5-HT2B (Ki = 3364 nM) and Sigma1 (Ki = 7408 nM).

Table S1. Whole-body *ex vivo* biodistribution study of [^{18}F]**7** in CD-1 mice. All data are mean ± SD, n = 3.

	5 min			15 min			30 min			60 min		
ID%/g	mean	SD	N	mean	SD	N	mean	SD	N	mean	SD	N
A. Brain	0.64	0.02	3	0.16	0.02	3	0.09	0.01	3	0.04	0.01	3
B. Blood	2.34	0.18	3	0.67	0.23	3	0.58	0.11	3	0.23	0.05	3
C. Muscle	3.13	0.21	3	0.91	0.17	3	0.73	0.22	3	0.19	0.04	3
D. Spleen	3.01	0.25	3	0.85	0.08	3	0.55	0.11	3	0.23	0.09	3
E. Heart	5.22	0.70	3	1.44	0.26	3	1.03	0.40	3	0.40	0.12	3
F. Lung	5.10	1.36	3	1.31	0.32	3	0.85	0.20	3	0.40	0.20	3
G. Pancreas	6.67	0.71	3	1.94	0.47	3	1.17	0.29	3	0.63	0.35	3
H. Stomach	1.91	0.32	3	0.99	1.00	3	0.90	0.55	3	0.63	0.37	3
I. Small Intestin	12.61	5.04	3	22.23	11.38	3	17.79	7.73	3	6.13	1.71	3
J. Kidney	8.47	0.70	3	3.60	0.41	3	2.13	0.32	3	0.85	0.29	3
K. Liver	26.23	2.87	3	13.97	1.18	3	11.29	3.84	3	6.54	2.53	3
L. Bone	1.60	0.30	3	0.61	0.11	3	0.43	0.06	3	0.28	0.14	3

2. HPLC radio-chromatograms

Semi-prep radio-HPLC chromatogram of [^{18}F]7

Column: Phenomenex Luna® 5 µm C18(2) 100 Å Prep Column (10 × 250 mm)

Mobile phase: CH_3CN-H_2O= 45%-55%, containing 0.1% NEt_3

flow rate: 5.0 mL/min

UV: 254 nm

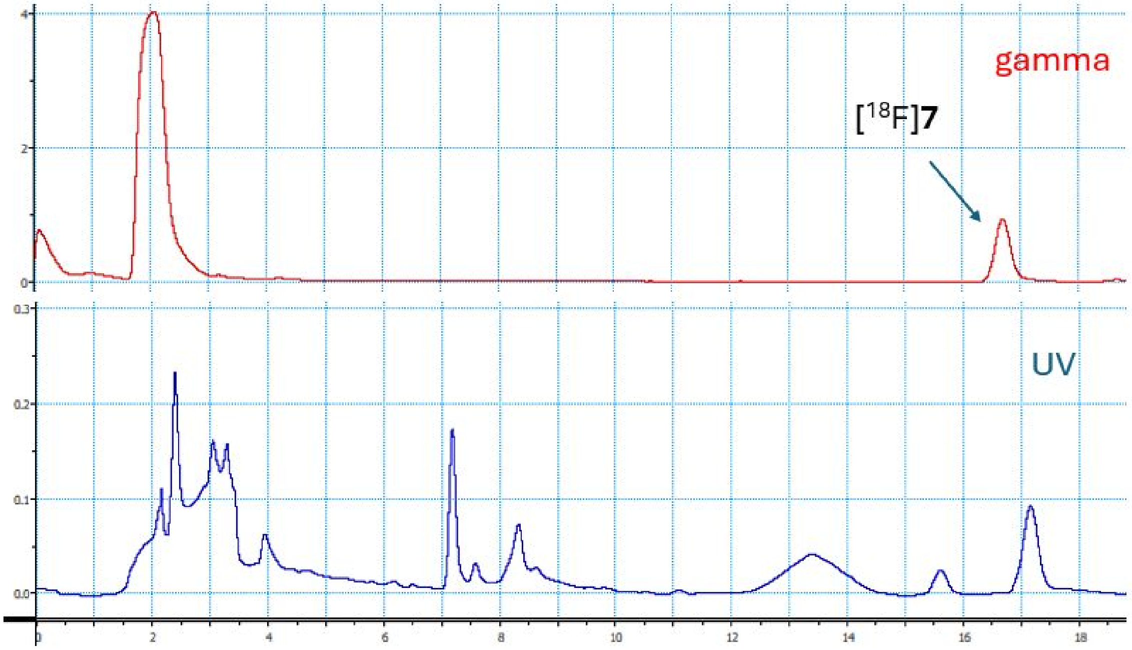

Analytical radio-HPLC chromatogram of [^{18}F]7

Chiral column: XBridge Prep Column (5 μm, 10 × 250 mm)

Mobile phase: CH_3CN-H_2O = 40%-60%, containing 0.1% NEt_3

flow rate: 1.0 mL/min

UV: 254 nm

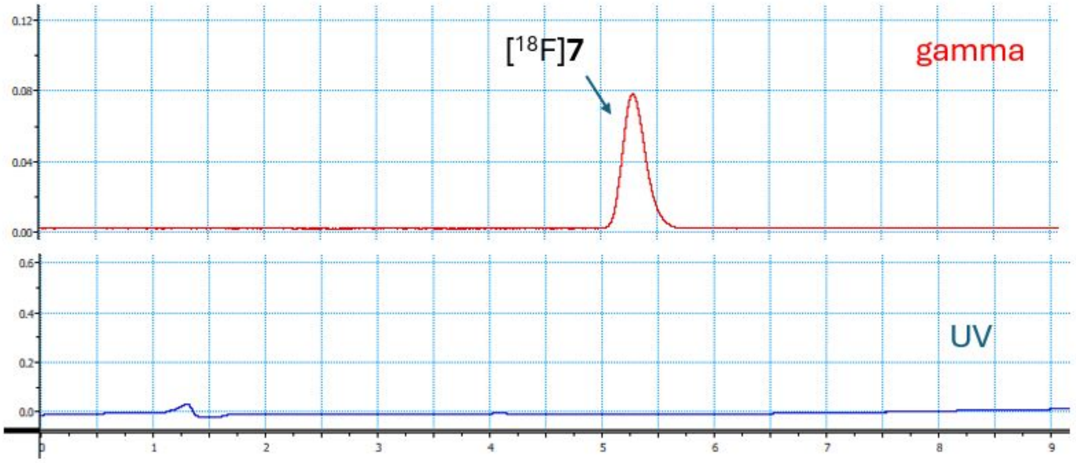

Co-injection of [^{18}F]7 with unlabeled 7

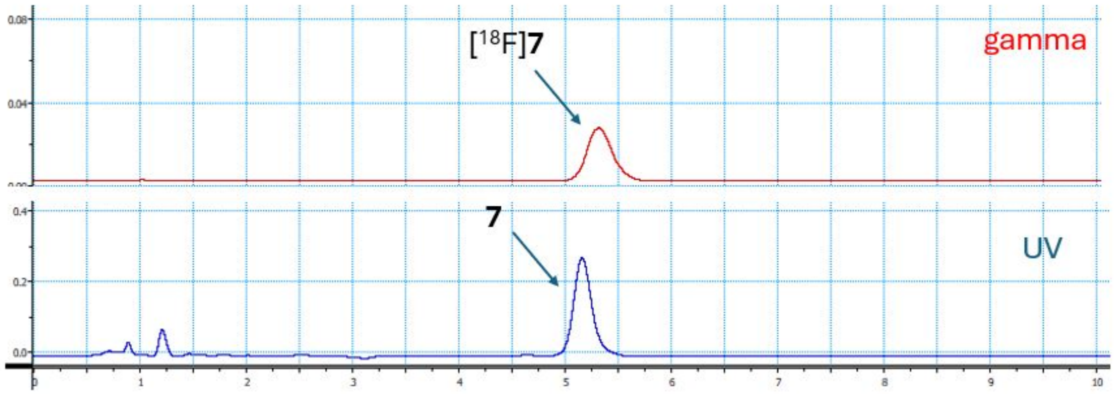

Calibration curve of compound 7

The specific activity of [^{18}F]**7** was calculated based on the calibration curve below.

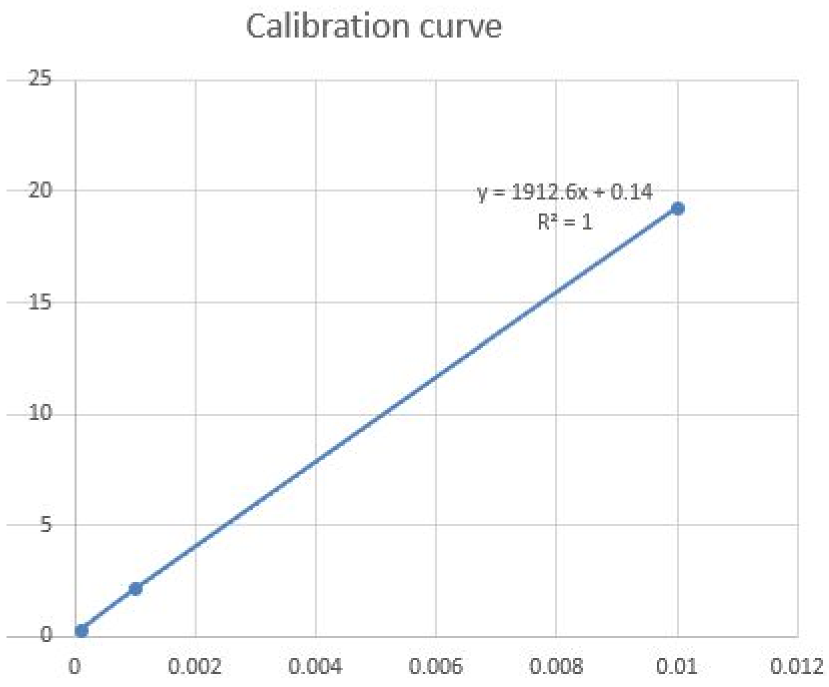

Radio-chromatograms of metabolite analysis

Radio-chromatograms of metabolite analysis in the brain

At 30 minutes post-administration of [^{18}F]**7**, 92% of [^{18}F]**7** remained unchanged in rat

brains (n = 2).

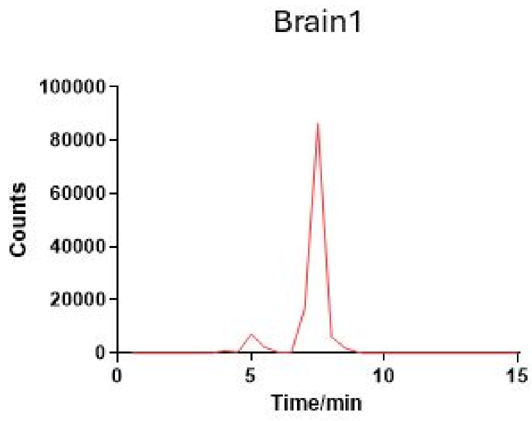

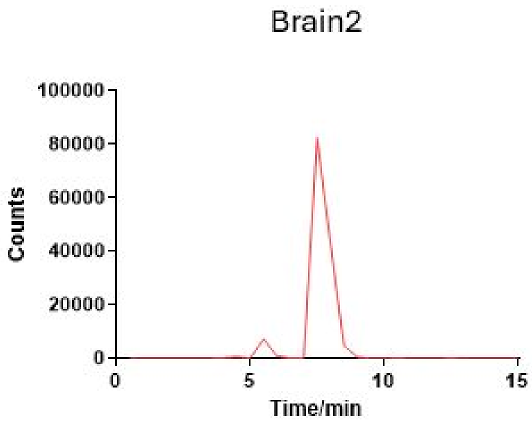

Radio-chromatograms of the metabolite analysis in plasma

At 30 minutes post-administration of [^{18}F]**7**, 67% of [^{18}F]**7** remained unchanged in rat plasma (n = 2).

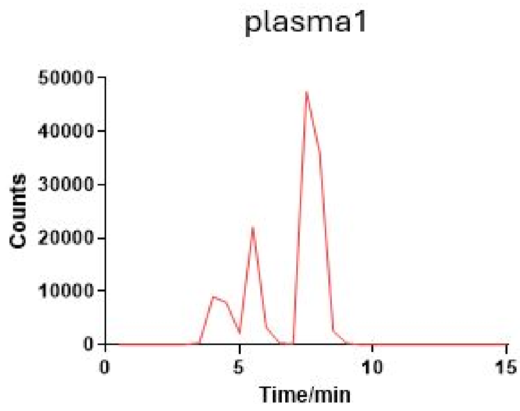

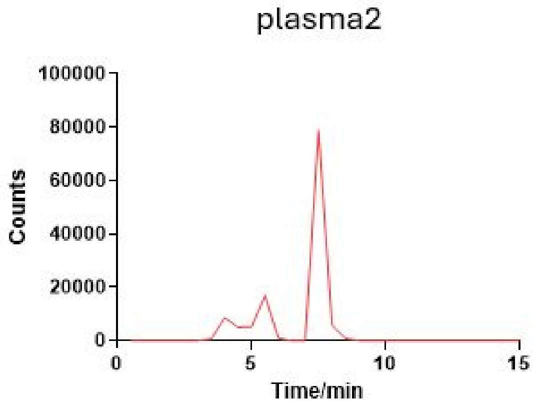

3. NMR spectra of isolated compounds

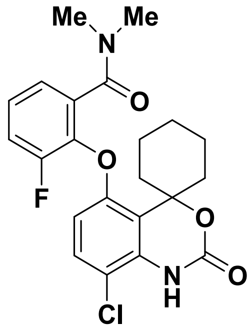

1 ^{1}H NMR
(400 MHz, $CDCl_3$)

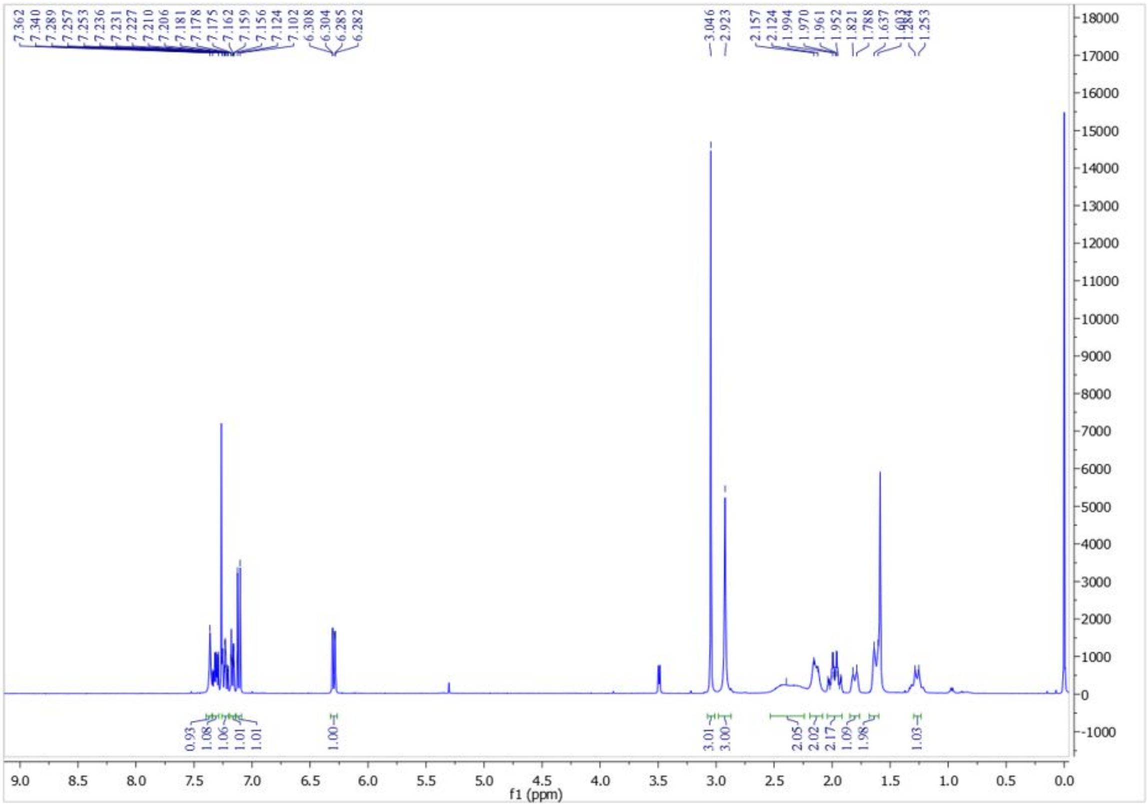

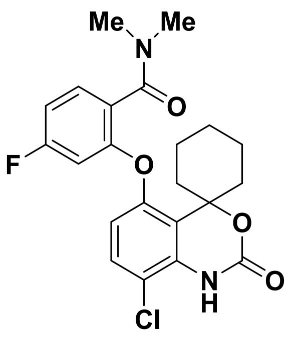

2 ^{1}H NMR
(400 MHz, $CDCl_3$)

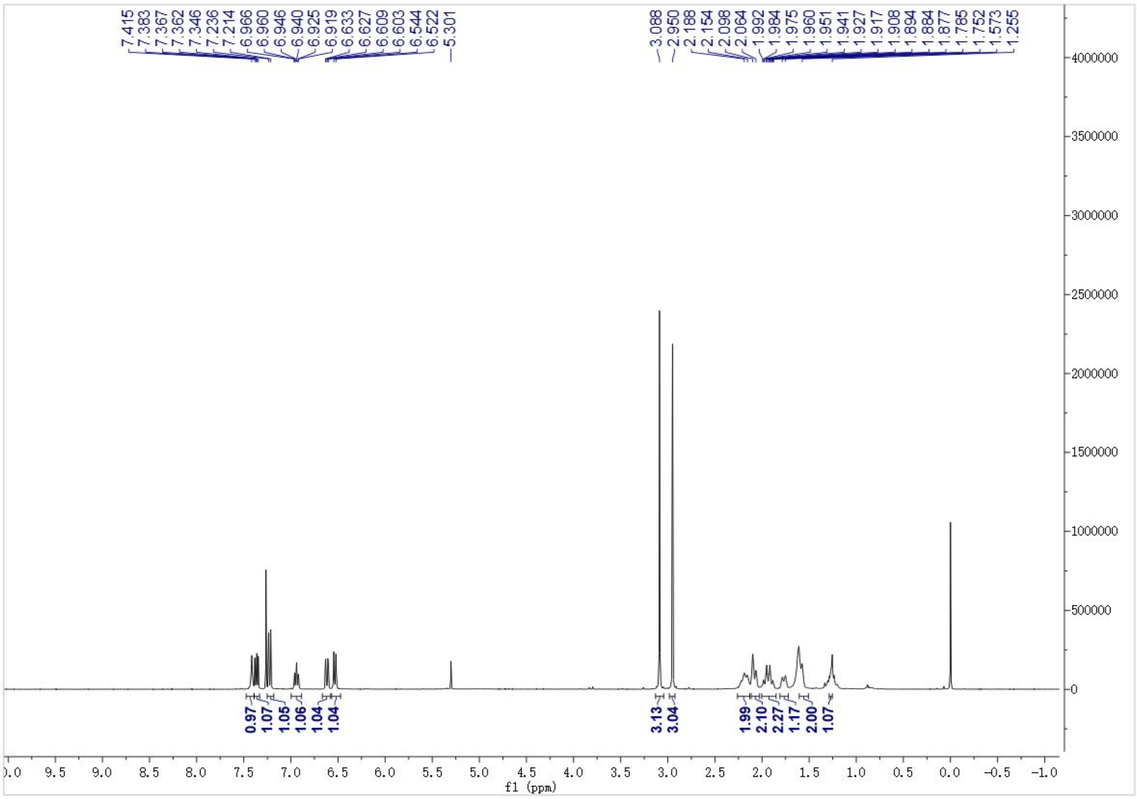

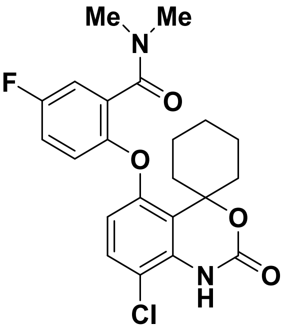

3 ^{1}H NMR
(400 MHz, $CDCl_3$)

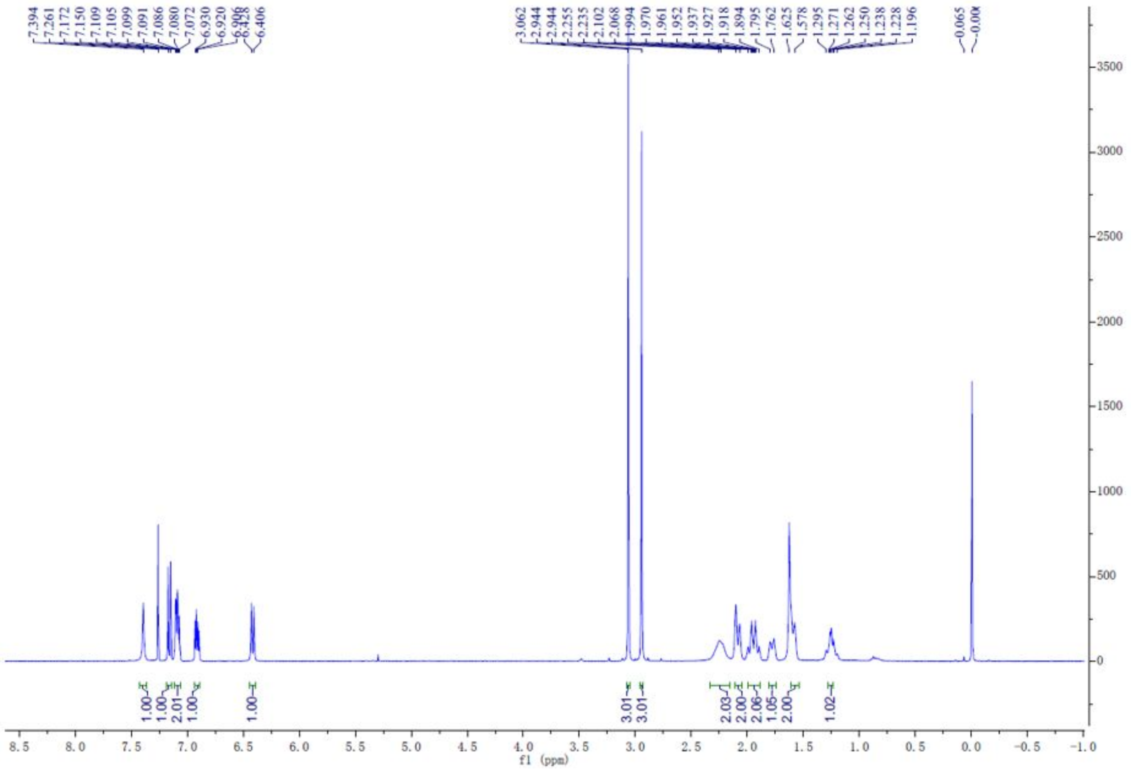

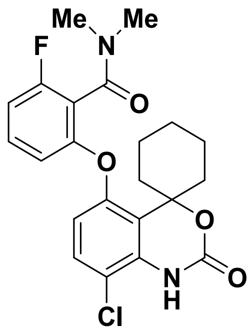

4 ^{1}H NMR
(400 MHz, $CDCl_3$)

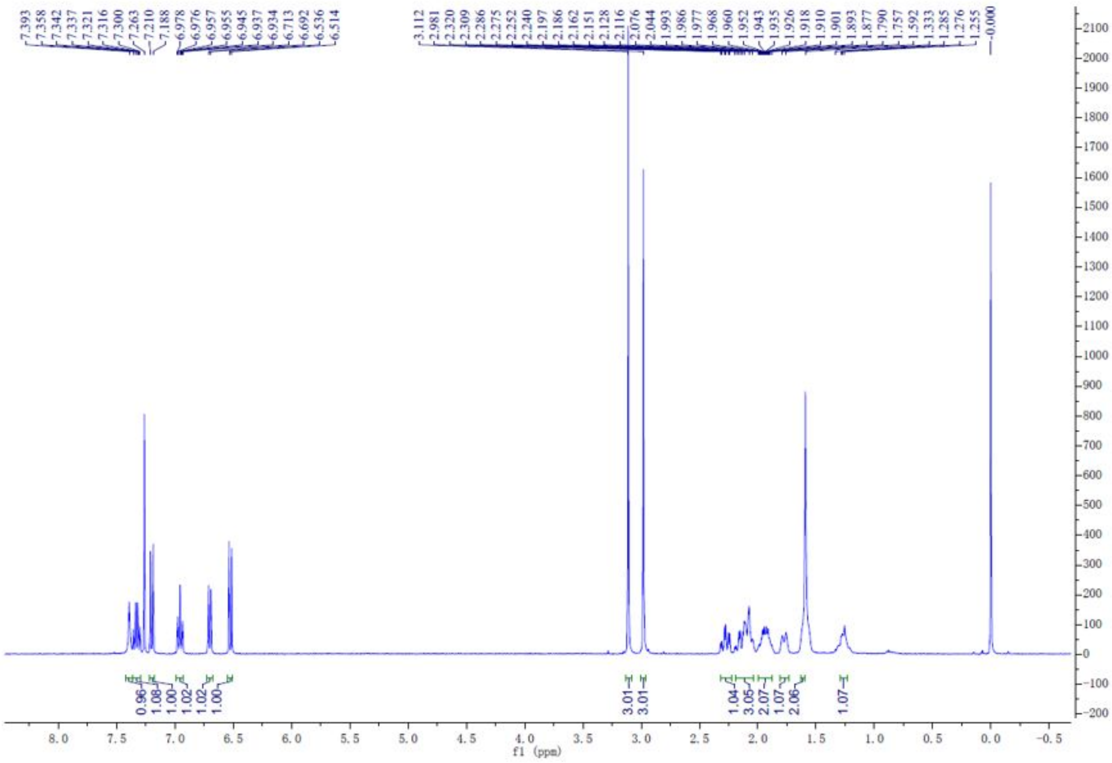

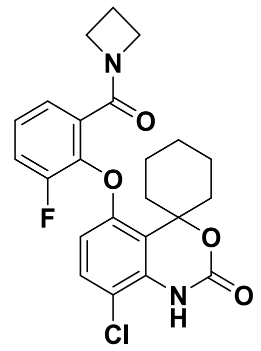

5 ^{1}H NMR
(400 MHz, $CDCl_3$)

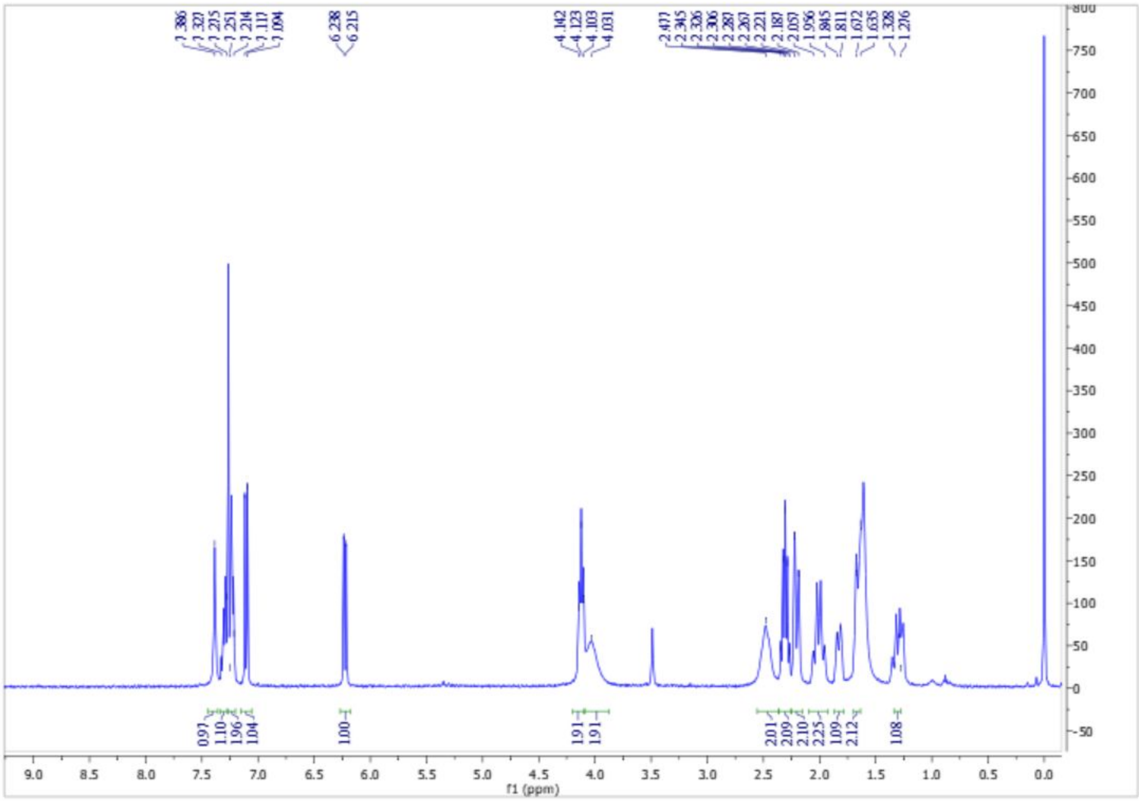

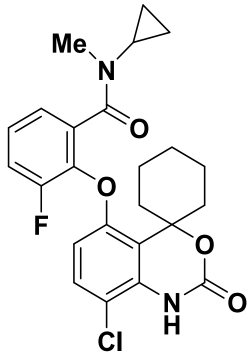

6 ^{1}H NMR
(400 MHz, $CDCl_3$)

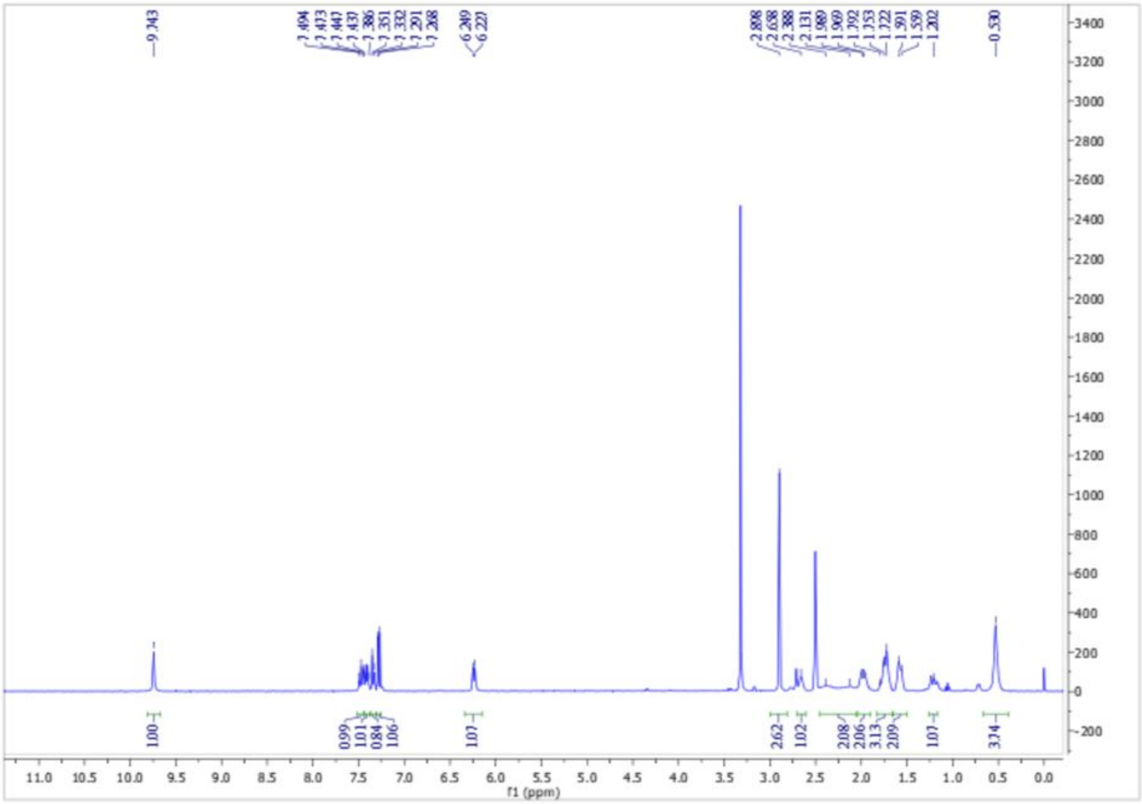

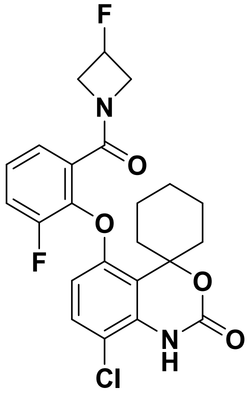

7 ^{1}H NMR
(600 MHz, $CDCl_3$)

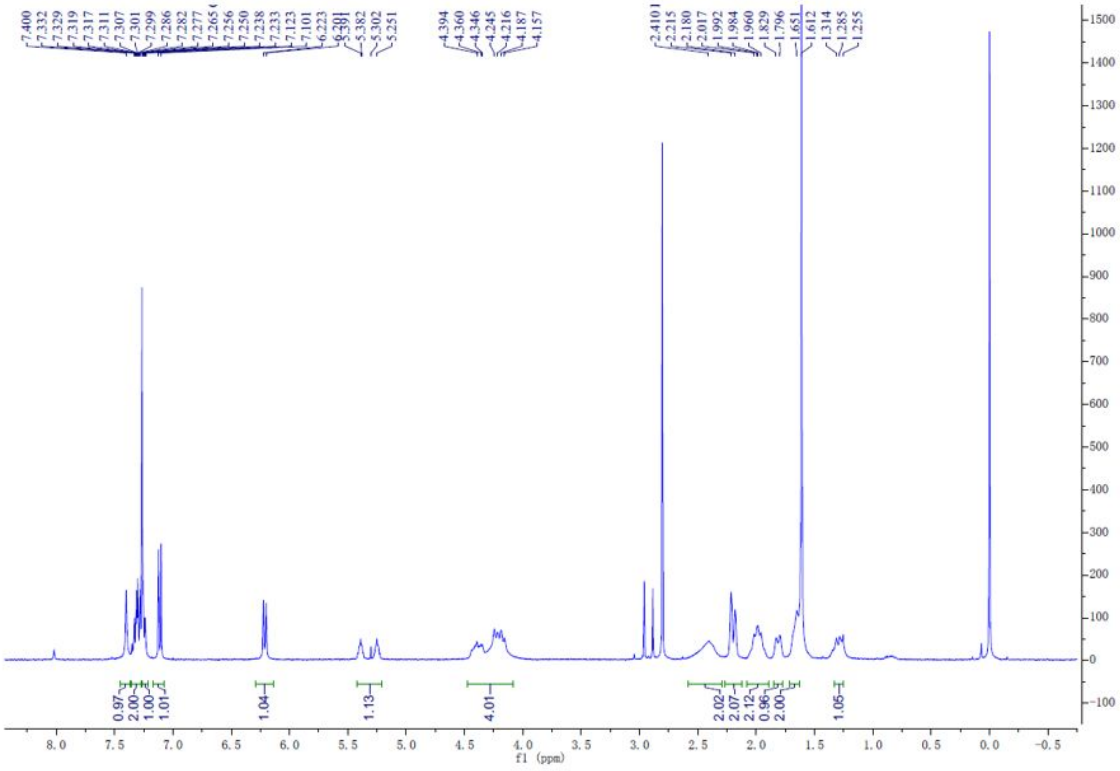

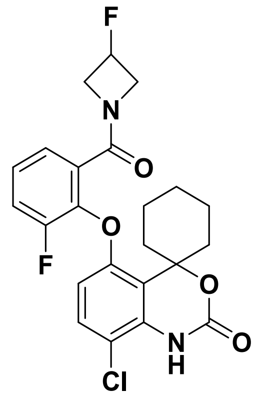

7 ^{13}C NMR
(150 MHz, $CDCl_3$)

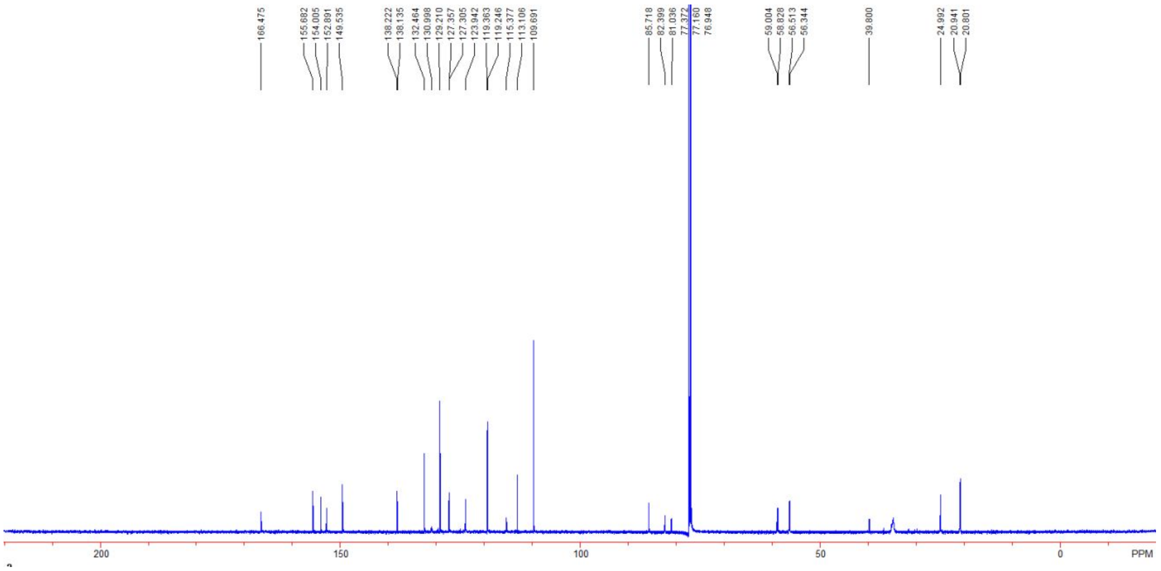

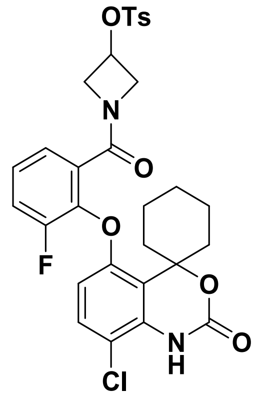

10 ^{1}H NMR
(600 MHz, $CDCl_3$)

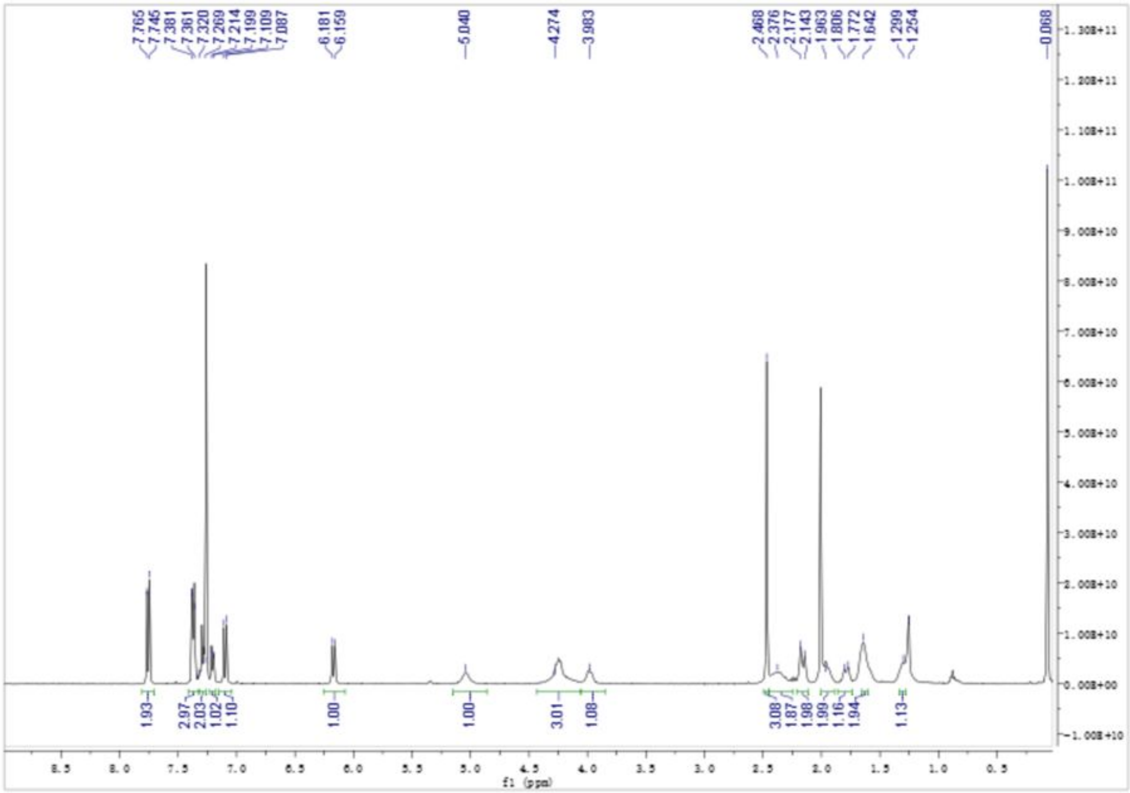

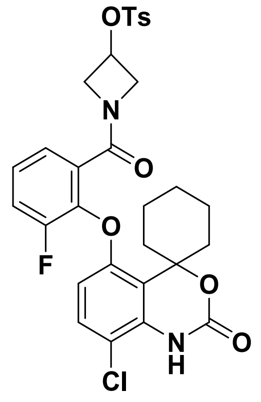

10 ^{13}C NMR
(150 MHz, $CDCl_3$)

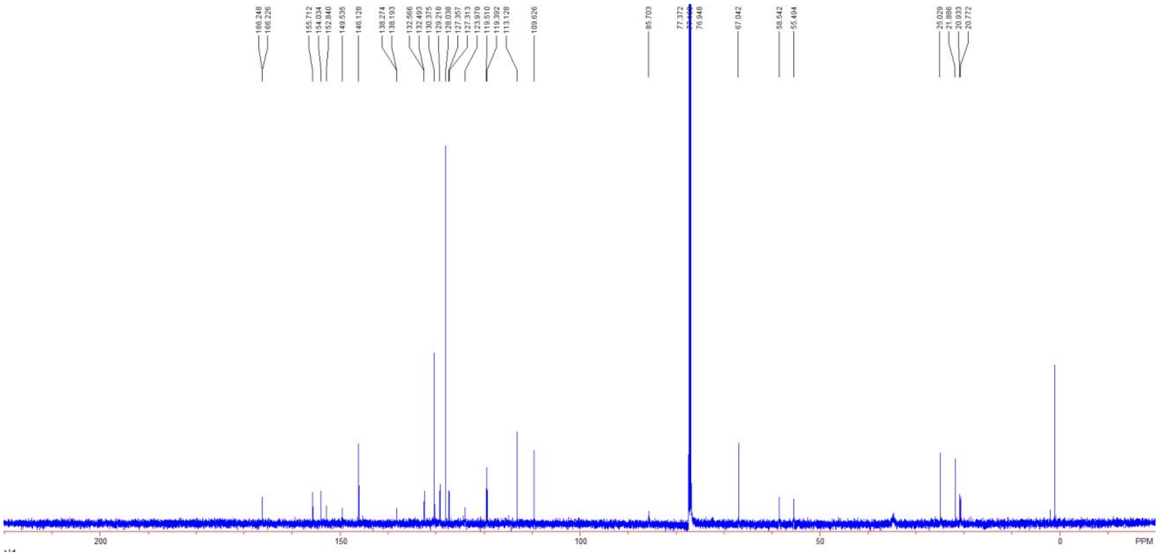

Supplement: Supplementary file 1 — mp4c01379_si_001.pdf [file mp4c01379_si_001.pdf]
